# Saturated fatty acids and total and CVD mortality in Norway: a prospective cohort study with up to 45 years of follow-up

**DOI:** 10.1017/S0007114524001351

**Published:** 2024-08-28

**Authors:** Erik Kristoffer Arnesen, Ida Laake, Marit B. Veierød, Kjetil Retterstøl

**Affiliations:** 1 Department of Nutrition, Institute of Basic Medical Sciences, University of Oslo, Oslo 0317, Norway; 2 Norwegian Institute of Public Health, Oslo, Norway; 3 Oslo Centre for Biostatistics and Epidemiology, Department of Biostatistics, Institute of Basic Medical Sciences, University of Oslo, Oslo, Norway; 4 The Lipid Clinic, Department of Endocrinology, Morbid Obesity and Preventive Medicine, Oslo University Hospital, Oslo, Norway

**Keywords:** Fatty acids, Saturated fat, CVD, Cohort study

## Abstract

The contribution of dietary saturated fatty acids (SFA) to cardiovascular disease (CVD) and mortality remains debated after decades of research. Few previous studies had repeated dietary assessments and power to assess mortality. Evidence for individual SFA is limited. In this large population-based cohort study, we investigated associations between intake of total and individual SFA and risk of total and CVD mortality. Adult residents (mean 41·1 years at baseline) in three Norwegian counties were invited to repeated health screenings between 1974 and 1988 (> 80 % attendance). We calculated cumulative average intakes of macronutrients from semi-quantitative FFQ. Median (interquartile range) intake of SFA was 14·6 % (12·8–16·6 %) of total energy (E%). Hazard ratios (HR) and 95 % CI were estimated using multivariable Cox regression models to assess total, CVD, ischaemic heart disease (IHD) and acute myocardial infarction (AMI) mortality. Among 78 725 participants, 28 555 deaths occurred during a median follow-up of 33·5 years, with 9318 deaths due to CVD. Higher intake of SFA (replacing carbohydrates) was positively associated with all mortality endpoints, including total (HR per 5 E% increment, 1·18; 95 % CI 1·13, 1·23) and CVD mortality (1·16; 95 % CI 1·07, 1·25). Theoretical isoenergetic substitution of SFA with carbohydrates or MUFA was associated with lower risk. Of individual SFA, myristic (14:0) and palmitic acid (16:0) were positively associated with mortality. In summary, dietary SFA intake was strongly associated with higher total and CVD mortality in this long-term cohort study. This supports policies implemented to reduce SFA consumption in favour of carbohydrates and unsaturated fats.

Diet is an important modifiable risk factor for mortality and the overall burden of non-communicable diseases, particularly cardiovascular disease (CVD)^(1)^. Current guidelines to prevent CVD promote predominately plant-based diets high in fruits and vegetables, whole grains, and foods high in unsaturated fats while reducing saturated fatty acids (SFA), red and processed meat, Na, and added sugars^(2)^.

Historically, prevention of CVD primarily focused on dietary fats, especially SFA^(3)^. The impact of dietary SFA on serum low density lipoprotein (LDL)-cholesterol, a causal risk factor for atherosclerosis, is extensively documented^(4)^. Guidelines generally recommended limiting SFA to < 10 % of total energy intake (E%), preferably replaced with polyunsaturated fatty acids (PUFA)^(5)^. However, some researchers have advocated against having this recommendation in dietary guidelines, arguing that the evidence linking SFA to CVD or mortality remains inconclusive^(6,7)^. While clinical trials support replacing dietary SFA with PUFA to prevent CVD^(8–10)^, results from observational studies are inconsistent. Previous systematic reviews of prospective cohort studies found no significant associations between SFA intake and total mortality or risk of coronary heart disease (CHD also known as ischaemic heart disease (IHD)), or stroke^(11–13)^. Mazidi et al. found a modest, positive association between SFA intake and CHD mortality, but not total nor CVD mortality^(14)^. In contrast, a recent systematic review commissioned by the World Health Organization (WHO) found a positive association between intakes of SFA and total mortality; cause-specific mortality was not assessed^(15)^.

Methodological issues in observational studies have likely played a role in generating conflicting evidence^(16–18)^. Many studies have suffered from small sample sizes or short follow-up periods, limiting the ability to identify significant associations with mortality. Furthermore, most studies have relied on a single baseline dietary assessment, which may not reflect cumulative exposure and thereby potentially attenuate associations^(19,20)^. Furthermore, the specific substitutions for SFA have often not been specified. The effects of reducing SFA intake may depend on the replacements chosen^(15,21)^, since replacing SFA with PUFA improves the lipid profile more than replacing it with carbohydrates^(4)^.

Individual SFA has distinct effects on blood lipids according to their carbon chain length^(4,22)^. Three of the quantitatively most prevalent SFA, lauric (C12:0), myristic (14:0) and palmitic acid (16:0), are presumably atherogenic as they increase levels of LDL-cholesterol and apolipoprotein B (apoB), whereas others are considered neutral^(22,23)^. Despite this, few studies have examined the associations between CVD and subtypes of SFA^(24–30)^. No previous cohort study has assessed the association between individual SFA and mortality from CVD or CHD, emphasising a need for further research^(31–33)^.

Prior modelling studies suggested that dietary changes, particularly reduced SFA consumption, contributed importantly to the decline in CVD incidence and mortality in Norway after the 1970s^(34,35)^. However, a direct assessment of long-term SFA consumption during this period and its association with CVD or total mortality in Norway has been lacking until now. Previous research from our group found an association between ruminant *trans*-fatty acids (TFA) intake and increased CVD mortality in a large population-based cohort study with a mean follow-up of 25·8 years^(36)^. In the present study, we aimed to examine the associations between dietary SFA and total mortality as well as deaths from CVD, IHD and acute myocardial infarction (AMI) in the same cohort, with an additional decade of follow-up. We accounted for changing SFA consumption over time, modelled the substitution of SFA for other macronutrients and assessed associations with individual SFA.

## Methods

### Study design and population

The Norwegian Counties Study is a population-based prospective study conducted in three Norwegian counties (Finnmark, Sogn og Fjordane, and Oppland) as a comprehensive CVD screening and prevention project^(37)^. Between 1974 and 1988, three screenings were performed, using the same procedures in each county, as previously described^(37,38)^. At screening I (1974–1976), all residents in the three counties aged 35–49 years (birth year 1925–1941), and a 10 % sample of 20–34-year-olds, were invited (*n* 65 624). The same birth cohorts were invited to screening II (1977–1983) and screening III (1985–1988), along with random samples from younger and older birth cohorts^(36,39)^. Attendance was above 80 % at all screenings (online Supplementary Table 1). In total, 92 234 individuals attended at least one screening.

Each screening included physical examinations, and a questionnaire covering lifestyle habits, medical history and marital status. Individuals with high CVD risk factors were referred to their general practitioners for follow-up.

Informed consent was obtained at the screenings from the participants but not recorded, according to common practice in research of that time. For the present analysis, ethical approval was obtained from the Regional Committee for Medical Research Ethics Norway (no. 2018/1302), the University of Oslo’s internal privacy protection commission and the Norwegian Institute of Public Health.

### Dietary assessment

At each screening, participants received a semi-quantitative food frequency questionnaire (FFQ) to be filled in at home and returned by mail. In Finnmark, the FFQ was only used in one municipality at screening I. The number of items varied from approximately sixty items in screening I to eighty in screening III, covering the frequency, types and amounts of foods, particularly foods that were important sources of fats in the Norwegian diet, as the interest was originally to elucidate associations with serum lipid patterns^(40)^. Foods included bread, bread spreads (including butter and types of margarine) and toppings, fats used in cooking and baking, milk (whole or skim), cheese, meat, fish, potatoes, and eggs.

Methods for calculating nutrient intakes from the FFQ have been described in detail previously^(36,41)^. Standard portion sizes were used to estimate amounts of foods^(42)^. Amounts of fats and spreads used on sandwiches were also assessed in the FFQ by using illustrated cubes representing different quantities. Nutrient calculations were performed using the Norwegian Food Composition Table from 1984 and 1991^(43)^. Fatty acids in Norwegian margarines and fats at the time of screening was provided by a Norwegian margarine producer (Denofa-Lilleborg), while dairy fat composition data were obtained from the Norwegian dairy cooperative Tine. Nutrient intake calculations were restricted to participants with no more than ten unanswered items on the FFQ. Of the participants who returned the FFQ, 93 %, 82 % and 84 % had sufficient data for nutrient calculations from screenings I, II and III, respectively. Furthermore, nutrient intakes were not considered valid if the total energy intake was < 2092 or > 16 736 kJ/d (in the lowest/highest 1 %).

Reproducibility of the FFQ was shown to have a median 81 % (range 50–98 %) agreement in a sample of 1445 women^(40)^. The FFQ were also compared with 24-h recall assessments conducted in a subset of participants, which showed acceptable agreement for ranking daily food intakes, such as milk and bread^(44–46)^. Mean energy and fat intakes calculated from the FFQ were approximately 80 % of those from the 24-h recall, but the differences in macronutrient proportions were minor^(44–46)^.

### Covariates

Participants were categorised according to smoking status and duration as follows: never, previous < 14 years, previous ≥ 14 years, current < 20 years and current ≥ 20 years. If a participant answered ‘never’ at one screening but had answered ‘previous’ or ‘current’ smoker on a previous screening, they were classified as previous smokers. Physical activity during leisure and work was assessed on the questionnaire with four levels for each type of activity, ranging from mostly sedentary to regular, vigorous exercise or heavy manual work (see Table 1 for full description)^(47)^. Education data were obtained from Statistics Norway and categorised according to the Norwegian Standard Classification of Education as no education, compulsory (1–9 years), upper secondary (10–12 years) and higher education (≥ 13 years).

**Table 1.** Characteristics of participants across quintiles of SFA at baseline (Numbers and percentages; Mean values and standard deviations)

			Quintile of SFA									
	Total		Q1		Q2		Q3		Q4		Q5	
	*n*	%	*n*	%	*n*	%	*n*	%	*n*	%	*n*	%
*n*	78 725		15 745		15 745		15 745		15 745		15 745	
Saturated fat, E%												
Mean	14·7		10·7		12·9		14·4		16·1		19·6	
sd	3·2		1·2		0·4		0·4		0·6		2·3	
Sex												
Male, *n* (%)	39 655	50·4	7941	50·4	7591	48·2	7633	48·5	7963	50·6	8527	54·2
Female, *n* (%)	39 070	49·6	7804	49·4	8154	51·8	8112	51·5	7782	49·4	7218	45·8
Age (years)												
Mean	41·1		41·9		41·4		41·0		40·4		40·5	
sd	7·2		7·2		7·1		7·1		7·4		7·4	
Higher education, *n* (%)	7918	10·1	2235	14·2	1857	11·8	1479	9·4	1223	7·8	1124	7·1
Married, *n* (%)	63 704	80·9	12 641	80·3	12 783	81·2	12 911	82·0	12 797	81·3	12 572	79·8
Sedentary leisure time activity, *n* (%)*	14 812	18·8	2631	16·7	2796	17·8	2960	18·8	3045	19·3	3380	21·5
Sedentary at work, *n* (%)†	17 673	22·4	4057	25·8	3877	24·6	3502	22·2	3294	20·9	2943	18·7
Current smoking, *n* (%)	34 695	44·1	5554	35·3	6215	39·5	7028	44·6	7660	48·7	8238	52·3
BMI (kg/m^2^)												
Mean	24·8		25·3		24·9		24·7		24·5		24·5	
sd	3·6		3·7		3·7		3·6		3·5		3·5	
BMI ≥ 30 kg/m2, *n* (%)	6299	8·0	1539	9·8	1370	8·7	1236	7·9	1063	6·8	1091	6·9
Previous MI, *n* (%)	393	0·5	166	1·1	84	0·5	60	0·4	45	0·3	38	0·2
Previous stroke, *n* (%)	144	0·2	45	0·3	30	0·2	23	0·1	19	0·1	27	0·2
Diabetes, *n* (%)	541	0·7	116	0·7	91	0·6	102	0·6	101	0·6	131	0·8
Treatment for hypertension, *n* (%)	3047	3·9	905	5·7	686	4·4	571	3·6	482	3·1	403	2·6
	Mean	sd	Mean	sd	Mean	sd	Mean	sd	Mean	sd	Mean	sd
Total cholesterol (mmol/l), mean (sd)	6·12	1·23	6·07	1·25	6·08	1·21	6·11	1·22	6·14	1·21	6·19	1·24
Non-HDL-cholesterol‡ (mmol/l), mean (sd)	4·70	1·26	4·68	1·27	4·66	1·26	4·71	1·27	4·75	1·27	4·75	1·25
Systolic blood pressure (mmHg), mean (sd)	133	17	133	17	132	17	132	16	133	17	133	17
Energy intake (kJ), mean (sd)	7100	2544	6418	2364	6841	2430	7150	2586	7401	2552	7690	2590
Total fat, E%	37·5	5·9	31·2	4·9	35·8	4·3	38·2	4·4	40·0	4·3	42·6	4·4
Cis-MUFA, E%	9·2	1·5	7·7	1·3	8·8	1·1	9·4	1·2	9·8	1·2	10·5	1·2
Cis-PUFA, E%	5·9	2·7	5·5	2·7	6·4	2·7	6·6	2·8	6·3	2·7	5·0	2·6
*n*-3 PUFA, E%	1·4	0·6	1·4	0·6	1·5	0·6	1·4	0·6	1·4	0·5	1·2	0·5
*n*-6 PUFA (18:2*n*-6), E%	4·6	2·4	4·1	2·3	4·9	2·4	5·2	2·4	4·9	2·4	3·9	2·2
TFA, E%	3·1	1·0	2·4	0·9	3·0	0·9	3·3	0·9	3·4	1·0	3·2	1·2
CHO, E%	47·2	5·6	52·8	4·8	48·9	4·1	46·8	4·2	45·1	4·2	42·6	4·5
Protein, E%	15·4	2·5	16·4	3·0	15·6	2·5	15·2	2·3	15·0	2·0	14·7	2·1

E%, energy percentage; MI, myocardial infarction; MUFA, monounsaturated fatty acids; PUFA, polyunsaturated fatty acids; TFA, *trans*-fatty acids; CHO, carbohydrates.

* Leisure physical activity categorised as sedentary (reading, watching TV and other sedentary work), moderate (walking, bicycling or other activity for min. 4 h/week), high (light sports, heavy gardening, etc.) and intensive (hard exercise and regular competitive sports several times a week)^(47)^.

† Occupational physical activity categorised as sedentary (primary sedentary work), walking (work requiring substantial walking), walking and lifting (substantial walking and lifting) and heavy manual labour^(47)^.

‡ Non-HDL-cholesterol only measured at screening II and III.

The participants were also asked about history of myocardial infarction, angina, stroke, diabetes mellitus, hypertension treatment or use of nitroglycerine; this was used to indicate presence of co-morbidities in this study. Measurements of weight, height, blood pressure and blood samples were taken at the screenings. Body mass index (BMI) was calculated (kg/m^2^). Non-fasting serum total cholesterol was measured non-enzymatically (screening I) and enzymatically (screening II and III) at Ullevaal Hospital’s central laboratory, Oslo, Norway, by the same staff using the same equipment for all counties. The non-enzymatically measured cholesterol was corrected to be compatible with enzymatic analyses^(48)^. Non-HDL-cholesterol, a proxy measure for atherogenic lipoproteins, was calculated by subtracting HDL-cholesterol from total cholesterol. HDL-cholesterol was only available from screening II and III. LDL-cholesterol could not be calculated since fasting triacylglycerol samples were not available^(49)^.

### Outcomes and follow-up

Underlying causes of death from death certificates were obtained through linkage to the national Cause of Death Registry, utilising the unique personal identification number assigned to all residents of Norway. Deaths from total CVD (diseases of the circulatory system, including IHD), IHD (which encompasses angina pectoris, AMI and other acute or chronic IHD) and AMI specifically were defined according to the European Shortlist for Causes of Death (COD-SL-2012), corresponding to the following codes from the International Classification of Diseases (ICD): CVD (ICD8:390–458; ICD9:390–459; ICD10: I00-I99), IHD (ICD8–9:410–414, 4140, 4143, 4148–9; ICD10: I20-I25) and AMI (ICD8–9:410–11; ICD10: I21–22). Follow-up started at a participant’s baseline screening, which we defined as the first screening in which our requirements regarding daily nutrient intakes were fulfilled, age ≥ 18 and < 65 years, the questions about smoking, physical activity, marital status and co-morbidities were answered, and measurement of BMI was obtained. Thus, start of follow-up for a participant may be later than the first attended screening. The participants were followed until death, emigration or 31 December 2018, whichever occurred first.

### Statistical analysis

All macronutrients were adjusted for energy by the energy density method and are reported as percentage of total energy intake (E%), excluding alcohol^(50)^. Pearson’s correlation coefficients were calculated for individual SFA to assess the degree of collinearity.

Longitudinal associations of SFA intake with serum total and non-HDL-cholesterol accounting for intra-individual correlations were examined by multilevel mixed-effects linear regression adjusted for age, sex, BMI, smoking, energy intake and other macronutrients with an individual-level random intercept, fit via maximum likelihood.

We estimated hazard ratios (HR) and 95 % CI using cause-specific multivariable Cox proportional hazards models with age as the timescale. To account for calendar-time effects, all models were stratified by birth cohort (< 1930, 1930–34, 1935–1939, 1940–1944, 1945–1949, 1950–1954, and ≥ 1955). Deaths from causes other than CVD, IHD or AMI were treated as censored from the time of death. Plots of smoothed scaled Schoenfeld residuals against age and observed Kaplan–Meier survival curves against predicted survival curves did not indicate violation of the proportional hazards assumption.

Associations between SFA intake and mortality were analysed using cumulative mean intake of SFA in quintiles, using the lowest quintile as reference. To reduce measurement error from intra-individual variation and to account for long-term intake, cumulative mean energy and nutrient intakes were calculated. Thus, intake at screening I was related to mortality outcomes until screening II, mean intake of screening I and II was related to mortality from screening II until screening III, and so on^(51)^. If only baseline intake data were available, it was carried forward for subsequent periods. We tested for trend by modelling cumulative mean SFA intake as a continuous variable. The shape of the dose–response associations was evaluated using restricted cubic splines with three knots placed at the 10th, 50th and 90th percentiles.

All covariates except sex were modelled as time-dependent and updated at each screening. We first adjusted for age (timescale) and sex. The multivariable-adjusted model included additional adjustments for potential confounding variables, guided by a directed acyclic graph (online Supplementary Fig. 1): cumulative mean energy intake (kcal/d, continuous), BMI (continuous), recreational and occupational physical activity, smoking status, attained education, marital status and self-reported co-morbidities (see above) at the time of each screening. We additionally adjusted for cumulative mean intakes of cis-MUFA, cis-PUFA, TFA and protein. This model estimates theoretically the effect of substituting energy from SFA with the same amount of energy from carbohydrates (CHO)^(50)^. Participants without complete measurements of all covariates from at least one screening were excluded from the analysis, as the level of missing data was < 2 % and likely not related to the outcomes. We performed additional analyses stratified on sex, BMI (< 18·5, 18·5–24·9, 25·0–29·9, ≥ 30 kg/m^2^) and presence of co-morbidities. Furthermore, multiplicative interactions between SFA intake (continuous) and sex, BMI (continuous), and presence of co-morbidities (dichotomous) were tested by using a likelihood ratio test.

Isoenergetic substitution analyses were conducted to examine the associations between SFA and mortality when energy from SFA was theoretically replaced with other macronutrients, by including all macronutrients in the multivariable model and calculating the differences between coefficients to estimate the HR and 95 % CI associated with the substitution^(52)^. We then modelled the replacement of 5 E% from SFA with carbohydrates (CHO), protein, MUFA, PUFA, *n*-6 PUFA and 1 E% replaced with *n*-3 PUFA.

Several sensitivity analyses were conducted, based on the multivariable model specified above. Potential reverse causality was explored by excluding the first 2 years of follow-up. We also assessed whether the associations were different between the first and last halves of the follow-up period. To address potential confounding by the overall diet quality, we additionally adjusted for the cumulative mean intake of potassium and vitamin C (in mg/1000 kcal), which are inversely associated with chronic disease, as indicators of a healthier diet^(53,54)^. In additional analyses, participants following special diets at the time of the screenings were excluded. These individuals had a significantly lower intake of SFA, which could indicate reverse causality. The presence of co-morbidities potentially has bidirectional relationships with nutrient intakes at both succeeding and preceding screenings, which could lead to time-varying confounding from disease-related factors. We therefore performed additional analyses without adjusting for co-morbidities. Furthermore, as BMI, physical activity and smoking could be a consequence of education, we repeated the analysis omitting education as a covariate. Finally, to assess the potential influence of using cumulative average intake of nutrients, we performed analyses using only the baseline dietary data.

All analyses were performed using Stata software, version 17 and 18 (Stata Corp.).

## Results

**Fig. 1.**
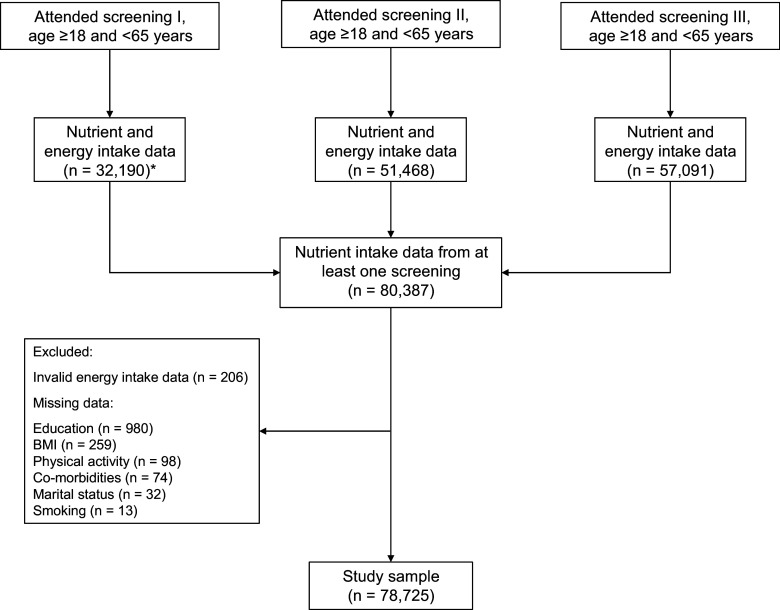
Flow chart of participants. * Dietary assessments at screening I were only used in two counties plus one municipality in the third county.

Of the 92 234 participants in screenings I–III, 80 387 were eligible for inclusion in this study (Fig. 1). Participants were ineligible if they did not fulfil the requirements of baseline at any of the attended screenings (see above). Attending participants with invalid nutrient intakes at all screenings or missing data on covariates at all screenings were also excluded (Fig. 1). Thus, 78 725 participants were included in the analyses.

### Participant characteristics

Mean age at baseline was 41·1 years (range 18·0–63·9). The mean intake of SFA at baseline across quintiles ranged from 10·7 to 19·6 E% (Table 1). Participants in the highest intake quintile were more likely to be male, younger, sedentary during leisure time and current smokers, compared with those in the lowest intake quintile (Table 1). In addition, they were less likely to be sedentary at work and have a BMI ≥ 30 kg/m^2^. Serum total cholesterol, total energy intake, as well as E% from MUFA and TFA, increased with intake of SFA. Participants not eligible for analyses of dietary intake were slightly younger, were more often males, current smokers, and sedentary during leisure time and work, and had marginally higher total cholesterol levels (online Supplementary Table 2).

The number of available dietary assessments was 30 991 in screening I, 50 172 in screening II and 55 954 in screening III. Intakes of both total fat and SFA decreased from screening I (1974–1976) through III (1985–1988). Specifically, mean total fat intake decreased from 38·7 to 35·4 E%, while SFA intake decreased from 15·7 E% to 13·2 E%.

Higher SFA intakes during follow-up was associated with higher total cholesterol and non-HDL-cholesterol levels (online Supplementary Fig. 2). Each 5 E% higher SFA intake was associated with a 0·22 (95 % CI 0·20, 0·24) mmol/l and 0·19 (95 % CI 0·17, 0·22) mmol/l higher total and non-HDL-cholesterol, respectively.

### Saturated fat intake and mortality

During a total of 2 489 121·1 person-years of follow-up (median 33·5 years, maximum 45 years), there were 28 555 deaths, including 9318 deaths from CVD, 4731 deaths from IHD and 3282 deaths from AMI. Cumulative mean intake of SFA was positively associated with total mortality (HR = 1·25; 95 % CI 1·18, 1·33, *P*_trend_ < 0·001, for highest *v*. lowest quintile, Table 2). A 5 E% increment in SFA intake was associated with an 18 % higher risk of total mortality (HR 1·18; 95 % CI 1·13, 1·23, *P*_trend_ < 0·001).

**Table 2.** Hazard ratios (HR) and 95 % CI of total, CVD, IHD and AMI mortality according to quintiles of cumulative average intakes of SFA and per 5 E% from SFA (*n* 78 725) (Hazard ratios and 95 % CI)

	Quintile of SFA intake (E%)											*P*_trend_ *
		Q2		Q3		Q4		Q5		Per 5 E%		
	Q1	HR	95 % CI	HR	95 % CI	HR	95 % CI	HR	95 % CI	HR	95 % CI	
Median intake, E%	11·4	13·2		14·6		16·1		18·7				
Person-years	576 383·17	549 945·42		517 158·42		466 037·25		379 596·83				
Total mortality												
Deaths, *n*	5971	6011		5974		5510		5089				
Crude incidence rate (deaths per 1000 person years)	10·36	10·93		11·55		11·83		13·40				
Sex-adjusted†	1 (ref.)	1·04	1·00, 1·07	1·08	1·04, 1·12	1·13	1·09, 1·17	1·25	1·20, 1·30	1·13	1·11, 1·16	< 0·001
Multivariable-adjusted‡	1 (ref.)	1·04	1·00, 1·08	1·06	1·01, 1·11	1·12	1·07, 1·18	1·25	1·18, 1·33	1·18	1·13, 1·23	< 0·001
CVD mortality												
Deaths, *n*	2019	1962		1917		1754		1666				
Crude incidence rate (deaths per 1000 person years)	3·50	3·56		3·71		3·76		4·38				
Sex-adjusted†	1 (ref.)	1·00	0·94, 1·06	1·00	0·94, 1·07	1·04	0·97, 1·11	1·11	1·04, 1·19	1·05	1·02, 1·09	0·005
Multivariable-adjusted‡	1 (ref.)	1·03	0·96, 1·10	1·04	0·96, 1·12	1·09	1·00, 1·19	1·25	1·12, 1·39	1·16	1·07, 1·25	< 0·001
IHD mortality												
Deaths, *n*	1005	967		975		912		872				
Crude incidence rate (deaths per 1000 person years)	1·74	1·76		1·89		1·97		2·30				
Sex-adjusted†	1 (ref.)	1·00	0·91, 1·09	1·04	0·95, 1·13	1·08	0·99, 1·19	1·14	1·04, 1·25	1·06	1·01, 1·11	0·027
Multivariable-adjusted‡	1 (ref.)	1·05	0·95, 1·16	1·10	0·99, 1·23	1·17	1·03, 1·32	1·31	1·13, 1·53	1·14	1·03, 1·26	0·014
AMI mortality												
Deaths, *n*	710	670		648		639		615				
Crude incidence rate (deaths per 1000 person years)	1·23	1·22		1·25		1·37		1·62				
Sex-adjusted†	1 (ref.)	0·98	0·88, 1·09	0·97	0·87, 1·08	1·07	0·96, 1·19	1·13	1·01, 1·26	1·05	0·99, 1·11	0·102
Multivariable-adjusted‡	1 (ref.)	1·03	0·92, 1·16	1·04	0·92, 1·18	1·17	1·01, 1·35	1·33	1·11, 1·59	1·15	1·02, 1·30	0·028

E%, energy percentage; IHD, ischaemic heart disease; AMI, acute myocardial infarction.

* *P* for linear trend.

† Cox proportional hazards regression stratified by birth cohort (< 1930, 1930–1934, 1935–1939, 1940–1944, 1945–1949, 1950–1954 and ≥ 1955) with age as timescale.

‡ Further adjusted for energy intake, BMI, physical activity, smoking habits, attained education, marital status, self-reported co-morbidities (i.e. history of myocardial infarction, stroke, angina or diabetes, treatment for high blood pressure or use of nitroglycerine), and energy from other types of fat and protein (in E%) and cholesterol (in mg/1000 kcal); the HR in this model can be interpreted as the associations with intakes of SFA at the expense of carbohydrates.

Intake of SFA was positively associated with CVD, IHD, and AMI mortality (Table 2). HRs per 5 E% higher intake of SFA at the expense of carbohydrates were 1·16 (95 % CI 1·07, 1·25, *P*_trend_ < 0·001) for CVD, 1·14 (95 % CI 1·03, 1·26, *P*_trend_ = 0·014) for IHD, and 1·15 (95 % CI 1·02, 1·30, *P*_trend_ = 0·028) for AMI.

**Fig. 2.**
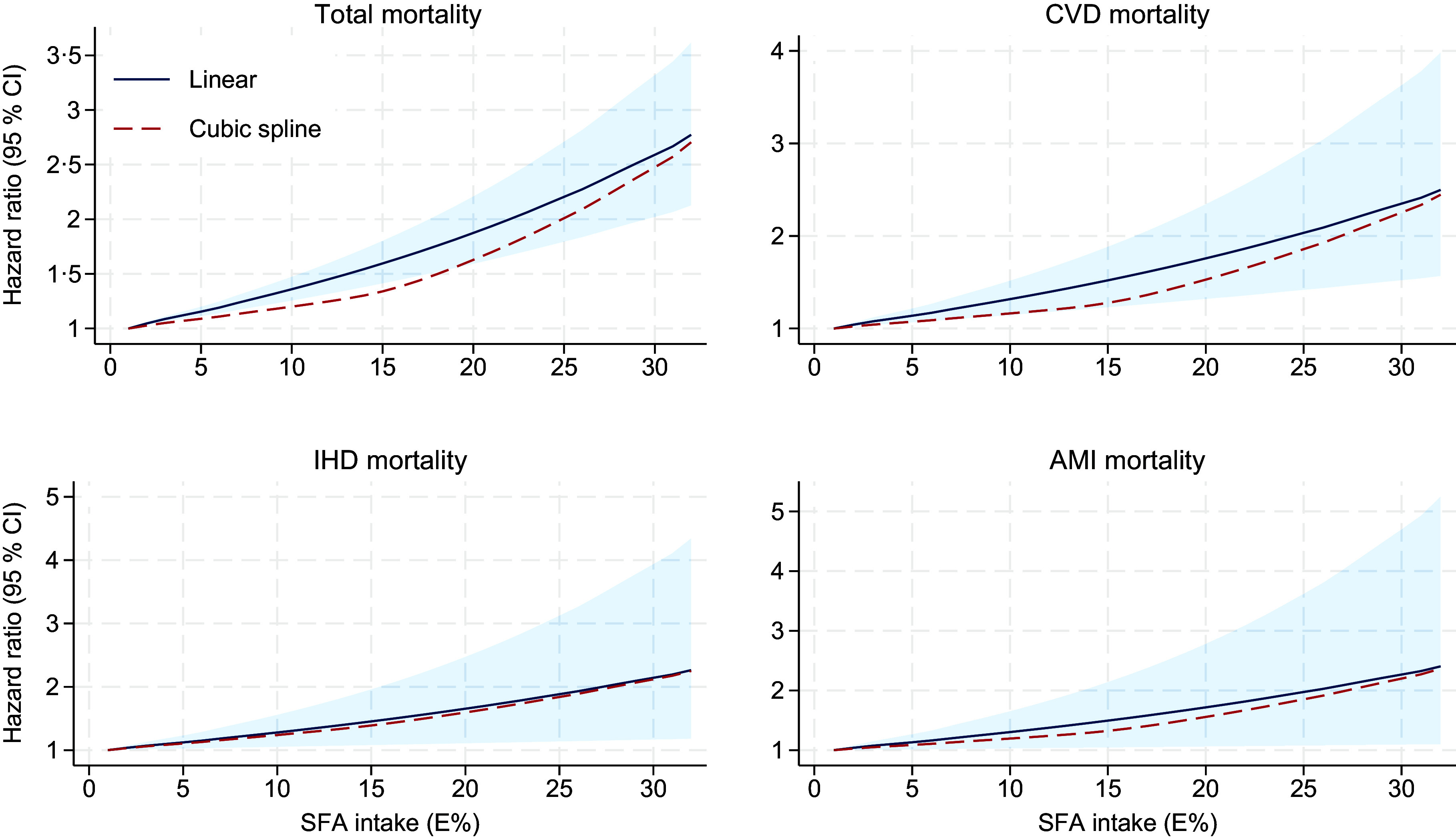
Multivariable dose–response relationship between E% from SFA and total and cause-specific mortality. The blue curve and shaded area denote hazard ratios and 95 % CI from the linear analysis, the dashed red line represents the restricted cubic spline model using three knots at the 10th, 50th and 90th percentiles. Cox proportional hazard models with age as timescale, stratified by birth cohort (< 1930, 1930–34, 1935–39, 1940–44, 1945–49, 1950–54 and ≥ 1955) and adjusted for sex, energy intake, BMI, physical activity, smoking habits, attained education, marital status, self-reported co-morbidities (i.e. history of myocardial infarction, stroke, angina or diabetes, treatment for high blood pressure or use of nitroglycerine), energy from other types of fat and protein (in E%) and cholesterol (in mg/1000 kcal); the HR in this model can be interpreted as the associations with intakes of SFA at the expense of carbohydrates. AMI, acute myocardial infarction; IHD, ischaemic heart disease.

Dose–response analyses using restricted cubic splines indicated a non-linear association between SFA and total mortality (*P*_non-linearity_ = 0·005), with a steeper increase in HR observed at intakes above 15 E% (Fig. 2). A similar pattern was seen for CVD (*P*_non-linearity_ = 0·09), whereas the dose–response curves were more linear for IHD and AMI (*P*_non-linearity_ = 0·76 and 0·49, respectively) (Fig. 2).

**Fig. 3.**
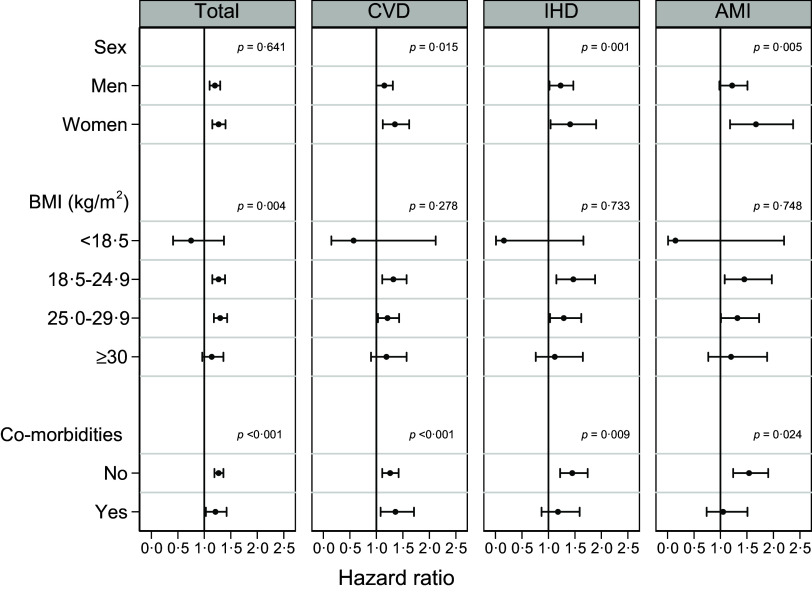
Hazard ratios and 95 % CI (horizontal lines) for total, CVD, IHD and AMI mortality for the highest *v*. lowest quintile of SFA in subgroups. Cox proportional hazard models with age as timescale, stratified by birth cohort (< 1930, 1930–34, 1935–39, 1940–44, 1945–49, 1950–54 and ≥ 1955) and adjusted for sex, energy intake, BMI, physical activity, smoking habits, attained education, marital status, self-reported co-morbidities (i.e. history of myocardial infarction, stroke, angina or diabetes, treatment for high blood pressure or use of nitroglycerine), energy from other types of fat and protein (in E%) and cholesterol (in mg/1000 kcal); the HR in this model can be interpreted as the associations with intakes of SFA at the expense of carbohydrates. *P*-values are from tests for interaction. AMI, acute myocardial infarction; IHD, ischaemic heart disease.

The association between SFA intake and total mortality was similar for males and females (*P*_interaction_ = 0·641). However, the association was stronger in females for CVD (*P*_interaction_ = 0·015), IHD (*P*_interaction_ = 0·001), and AMI (*P*_interaction_ = 0·005) mortality (Fig. 3 and online Supplementary Table 3). For all outcomes, the association with SFA was stronger among persons with BMI 18·5–24·9 kg/m^2^ than among those with BMI ≥ 30 kg/m^2^ (Fig. 3 and online Supplementary Table 4). Finally, the associations were stronger among persons with no co-morbidities for all outcomes except CVD (Fig. 3 and online Supplementary Table 5).

#### Isoenergetic substitution analyses

**Fig. 4.**
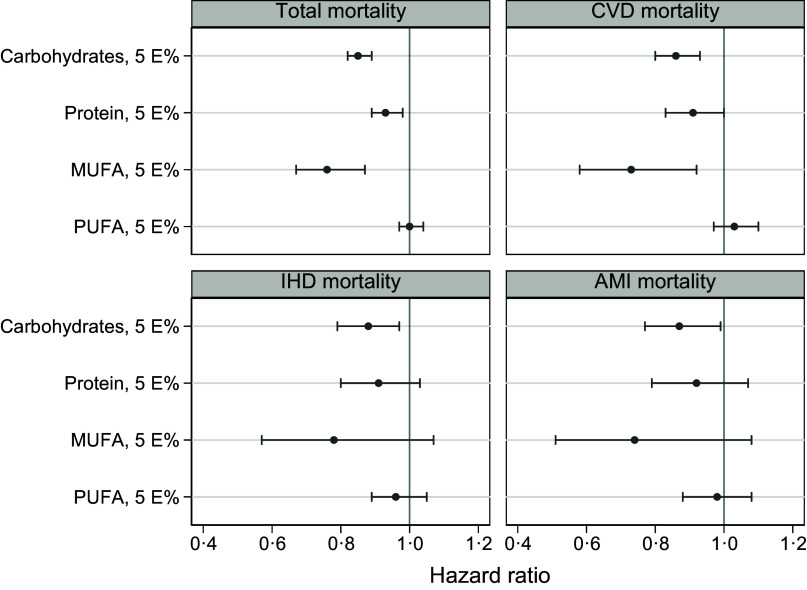
Hazard ratios with 95 % CI for total, CVD, IHD and AMI mortality according to isoenergetic substitution analyses modelling substitutions of 5 E% carbohydrates, protein, MUFA and PUFA for SFA. Cox proportional hazard models with age as timescale, stratified by birth cohort (< 1930, 1930–34, 1935–39, 1940–44, 1945–49, 1950–54 and ≥ 1955) and adjusted for sex, energy intake, BMI, physical activity, smoking habits, attained education, marital status, self-reported co-morbidities (i.e. history of myocardial infarction, stroke, angina or diabetes, treatment for high blood pressure or use of nitroglycerine), energy from other macronutrients (depending on type of substitution) and cholesterol (in mg/1000 kcal). AMI, acute myocardial infarction; IHD, ischaemic heart disease.

Each 5 E% higher intake of CHO or MUFA at the expense of SFA was associated with lower total mortality (HR 0·85; 95 % CI 0·82, 0·89 for CHO and 0·76; 95 % CI 0·67, 87 for MUFA) (Fig. 4 and online Supplementary Table 6). There was no association when increasing intake of total PUFA at the expense of SFA, although a small inverse association was observed with isoenergetic replacement of 5 E% from SFA with linoleic acid (HR 0·90; 95 % CI 0·86, 0·95). Similar associations were observed for CVD, IHD and AMI mortality. Substituting overall PUFA, linoleic acid or *n*-3 PUFA for SFA did not show associations with CVD, IHD or AMI mortality (online Supplementary Table 6).

### Individual SFA and mortality

Approximately 90 % of the total SFA intake was derived from 12:0–18:0. Strong correlations were found between the individual SFA (*r* = 0·65–0·93; online Supplementary Table 7). All individual SFA were positively associated with total mortality. HRs (95 % CI) for total mortality were 1·12 (95 % CI 1·06, 1·18) for 4:0–10:0, 1·17 (95 % CI 1·11, 1·23) for 12:0, 1·22 (95 % CI 1·15, 1·28) for 14:0, 1·24 (95 % CI 1·16, 1·34) for 16:0, and 1·18 (95 % CI 1·08, 1·29) for 18:0 (*P*_trend_ < 0·001 online Supplementary Table 8). On the other hand, 12:0 and 18:0 were not consistently associated with CVD, IHD, and AMI mortality (0·008 ≤ *P*_trend_ ≤ 0·375 and 0·104 ≤ *P*_trend_ ≤ 0·258, respectively; online Supplementary Table 8). For 16:0, the most abundant SFA, the associations were similar as those observed with total SFA (HR (95 % CI) 1·24 (95 % CI 1·09, 1·40) for CVD, 1·33 (95 % CI 1·12, 1·59) for IHD, and 1·26 (95 % CI 1·02, 1·56) for AMI mortality).

Theoretical isoenergetic substitution of 12:0–18:0 with carbohydrates, protein, MUFA, total PUFA and linoleic acid was associated with lower total mortality (*P* < 0·001–0·011; online Supplementary Table 9).

### Sensitivity analyses

When excluding the first 2 years of follow-up, the results for the associations with total SFA were practically identical (online Supplementary Table 10). Regarding total, CVD, and IHD mortality, the HRs tended to be stronger during the first half of the follow-up time than the second half (online Supplementary Table 11). The estimates were slightly strengthened when excluding participants that reported being on a diet (online Supplementary Table 12). When not adjusting for co-morbidities, the estimates were marginally strengthened (online Supplementary Table 13). The results were also almost identical when not adjusting for education (online Supplementary Table 14). Additional adjustment for vitamin C and potassium intake (available from 73 045 participants) slightly attenuated the estimates, although the HRs were still positive (online Supplementary Table 15). Using only the baseline nutrient intake instead of cumulative updated averages greatly attenuated the HRs (online Supplementary Table 16).

## Discussion

In this large population-based cohort with repeated measures of diet, we found a positive association between intake of SFA and total mortality and mortality from CVD, IHD and AMI over four decades of follow-up. Isoenergetic substitution analyses indicated that replacement of SFA with carbohydrates and MUFA was associated with significantly reduced risks. Among individual types of SFA, the associations were generally strongest and most consistent with myristic (14:0) and palmitic acid (16:0), the most abundant SFA.

### Comparison with prior literature

Regarding the association between dietary SFA intake and CVD mortality, results from previous cohort studies have been mixed. In a meta-analysis of three cohort studies, de Souza et al. reported no association between SFA intake and CVD mortality^(13)^. The lack of specification of replacement nutrients in the included studies complicated the comparison of higher *v*. lower intakes of SFA. More recently, Reynolds et al. found a higher risk of all-cause mortality in the highest *v*. lowest category of SFA intake, but no significant dose–response trend^(15)^.

In two pooled analyses of cohort studies, no associations with CHD and CHD mortality was found when SFA were isoenergetically substituted with carbohydrates^(55,56)^. Interestingly, in our present study, the theoretical substitution of SFA with carbohydrates was associated with lower mortality. This discrepancy is likely related to different sources of carbohydrates across cohorts. The diet of our study population was characterised by a relatively high intake of bread and potatoes and low intake of sugar-sweetened beverages^(45,46,57)^. Jacobs et al. observed an inverse association between the intake of whole grain bread and total, CHD, and CVD mortality in this cohort^(58)^, consistent with the overall literature on whole grains^(59)^. A different population with different sources of carbohydrates may have yielded different results. The largest cohort study to date, covering 129 328 total deaths, also reported positive associations between SFA intake and both total and CVD mortality when SFA was isoenergetically compared with carbohydrates, MUFA and *n*-6 PUFA^(60)^.

Randomised controlled trials (RCTs) have shown reduced risk of CHD events with the replacement of SFA with PUFA^(8,^ ^9)^. However, these RCTs were not powered to assess total or cause-specific mortality risks^(8,10)^. We did not find consistent associations between theoretical substitutions of SFA with total PUFA and CVD mortality, in contrast to previous cohort studies^(55,56)^. This could be related to the sources of PUFA in the study population, where margarines were a major source but also contained high levels of TFA and SFA^(36)^. Even after adjusting for TFA, it is possible that some of the *α*-linolenic acid in margarines may have been *trans* isomers^(60–62)^. The observed inverse association with total and CVD mortality when SFA was substituted with MUFA is in agreement with previous studies, although the source of MUFA (e.g. animal *v*. vegetable) may matter^(15,52)^. Considering that red meat and high-fat dairy products are high in both SFA and MUFA, our substitution analysis might reflect the replacement of SFA with non-animal sources of MUFA^(21,63)^.

Conflicting associations in some studies are conceivably due to different food sources of fatty acids, in addition to methodological approaches^(25,26,64,65)^. Some studies have been conducted in populations with a low SFA intake, well below typical intakes in Western diets, which may yield different associations and limit generalisability. Most studies to date have only assessed dietary intakes reported at baseline, which might not accurately reflect long-term intakes. In contrast, studies using several repeated dietary assessments and long durations of follow-up have found a lower risk of mortality associated with substitution of SFA with PUFA and carbohydrates from whole grains^(21,52,66)^.

No prior investigations have explored associations between individual SFA and CVD, IHD, or AMI mortality. Some previous studies indicated associations between 12–18:0 and CHD^(29,67,68)^. This was also found in a large study with repeated assessment of intake, where substitution with PUFA, MUFA, whole grains or plant protein was associated with lower risk^(24)^. The 12–18:0 were not associated and incident myocardial infarction in two cohorts from Denmark and the UK without repeated assessments^(27)^. In a Dutch cohort, only higher intake of 16:0 was associated with higher risk of CHD^(26)^.

### Potential mechanisms

The primary mechanism relating SFA to CVD is their adverse effects on serum LDL-cholesterol and apoB levels^(8,69)^. Elevated LDL-cholesterol is a causal risk factor for CVD especially when present from a young age, contributing to the initiation and development of atherosclerosis^(70)^. Previous cohort studies often adjusted for serum cholesterol when assessing the associations between SFA and CVD, which could attenuate the total effects of SFA. Effects on LDL-cholesterol is also a plausible explanation for the associations specifically observed with intake of 14:0 and 16:0^(4,22)^. 12:0 also raises LDL-cholesterol, but the general lack of independent associations in this study may be due to lower intake levels. Intake of 18:0 did not show clear associations with CVD, IHD and AMI mortality, possibly reflecting its neutral effect on blood lipids^(4)^. However, absence of associations should be cautiously interpreted due to the collinearity with other SFA^(24)^. As pointed out in the recent WHO guideline, ‘recommendations for individual SFA may be of limited utility to end users and difficult to implement’^(33)^.

Our subgroup analyses indicated that the associations between SFA and CVD, IHD, and AMI mortality were stronger in females than in males. This difference has also been observed in some previous cohorts^(28,71–73)^. While some trials have found higher blood lipid responses to SFA in males^(74)^, a recent systematic review found no significant heterogeneity^(4)^. The apparent interaction may have been due to more accurate dietary measurements in females^(71)^ or different diet qualities. The associations were weaker in those with BMI ≥ 30 kg/m^2^. It has been suggested that obesity attenuates the blood lipid response to changes in SFA and dietary cholesterol intakes due to increased inflammation and insulin resistance^(75–77)^. Alternatively, the apparently weaker associations could be affected by a different baseline risk or different background diets. Further, there may have been more misreporting in this group, which could attenuate the associations or reduce statistical power^(78,79)^. The high prevalence of obesity in today’s population warrants further examination of potential effect modifications. In the present study, the null associations between SFA and IHD and AMI mortality in the group with self-reported co-morbidities likely result from few participants (less than 10 % of the total population) and the possibility of reverse causality due to recent dietary changes.

Besides fatty acids *per se,* the associations may also be related to properties of their food sources. The top sources of SFA were milk and dairy products, margarine and butter, meat and offal^(45,46)^. At that time, milk was mostly full-fat milk, which has been associated with increased mortality and CVD^(80–82)^. On the other hand, replacing SFA from meat with dairy products might reduce risk, but more research is needed on specific foods and fats within dietary patterns^(83)^.

### Strengths and limitations

Strengths of this study include a large sample size, repeated measurements, a long follow-up period, a high response rate and follow-up through linkage with a national registry. The population was unselected and relatively young and healthy at baseline, reducing the risk of reverse causality and survivorship bias. Inclusion of individuals reporting co-morbidities, such as a history of myocardial infarction or stroke, was accounted for in the analyses. The results were also robust to multiple sensitivity analyses.

During the 1970s and 1980s, there was a considerable decline in the consumption of whole milk (semi-skimmed milk was introduced in 1984), margarine, and butter among the Norwegian population^(35)^. Throughout this period, the use of margarine also underwent changes towards more soft and less hard types, in adherence with official recommendations. Repeated measurements of intake were therefore crucial to reduce exposure misclassification and regression dilution bias. Accounting for long-term intake is also aetiologically relevant, since elevated intakes of SFA may have cumulative effects over time, analogous to the impact of LDL-cholesterol burden on CVD risk^(51,70)^. A further strength was access to detailed information from the margarine industry regarding the composition of margarines used (including TFA), which would not be available with more modern food composition data.

However, the study also has several limitations. Diet was self-reported, and the FFQ primarily aimed to capture fat intake, potentially leading to the underestimation of other nutrients. Comparisons with 24-h recall indicated that the FFQ underestimated the total energy intake by 17–20 %, particularly from vegetables and fruits^(45)^. However, the macronutrient densities (E%) were concordant^(45,46)^. Whether the intake of non-reported foods varied with SFA intake is unknown. Despite this, we adjusted for total energy intake and other factors related to underreporting, such as age, sex, and BMI^(20,84)^. Expected associations were found between SFA and total cholesterol and non-HDL-cholesterol. While we consider repeated measurements a strength, we recognise that misclassification of SFA intake could still be possible since there were only three dietary assessments with a considerable time gap between the last assessment and the end of follow-up. Most of the changes in fat and SFA consumption at the population level in Norway occurred before 1990, but the lack of subsequent dietary data precluded us from verifying this in the cohort^(34)^. Nutrient intakes at all screenings were calculated from the 1984 and 1991 food composition tables, which largely relied on older nutrient analyses, although there could have been some differences in the composition of foods consumed in the 1970s. Earlier versions did not have data on saturated and unsaturated fats.

We could not assess the intake of less common SFA, such as odd-chain SFA. Further, due to shared food sources, the high correlations between individual SFA posed challenges for analysis and interpretation of their independent contributions, as noted previously^(24)^. To partly address collinearity, we combined 12:0–18:0 into a composite variable.

Residual confounding must also be considered. First, we could not differentiate between SFA from animal and vegetable sources. Second, we could not adjust for alcohol consumption, which according to 24-h recall accounted for about 1 E%^(45,46)^. Third, socio-economic status was indicated only by education. Fourth, data on lipid-lowering medication use during follow-up were unavailable, potentially affecting the risk associated with high SFA if participants with higher intakes, and thus higher serum cholesterol, were more likely to become eligible for statins in the last part of the observation period. Due to changes in diets and risk profiles, it is unclear if the results of this study may fully generalise to today’s population.

The Norwegian Causes of Death Registry has high coverage and quality^(85)^, but misclassifications in death certificates do occur. Diagnostic criteria for IHD have also evolved over the years. A previous study found high validity for IHD deaths in a Norwegian cohort compared with autopsy data^(86)^. Potential outcome misclassification was unlikely related to SFA intake.

### Conclusion

We found a positive association between intake of SFA and total mortality and cardiovascular mortality over four decades of follow-up. Our study supports dietary recommendations to reduce SFA consumption and replace them with unsaturated fats and/or carbohydrates. Furthermore, the study indicates that long-term dietary assessments may be necessary to detect associations between intake of SFA and total and CVD mortality. Further research should consider the complexities of food sources of SFA and potential interactions with various risk factors.

## Supporting information

Arnesen et al. supplementary material Arnesen et al. supplementary material

## Supplementary material

For supplementary material accompanying this paper visit https://doi.org/10.1017/S0007114524001351

## References

[1] Dong C , Bu X , Liu J , et al. (2022) Cardiovascular disease burden attributable to dietary risk factors from 1990 to 2019: a systematic analysis of the Global Burden of Disease study. Nutr Metab Cardiovasc Dis 32, 897–907. 35067445 10.1016/j.numecd.2021.11.012

[2] Arnett DK , Blumenthal RS , Albert MA , et al. (2019) 2019 ACC/AHA guideline on the primary prevention of cardiovascular disease: a report of the American College of Cardiology/American Heart Association Task Force on Clinical Practice Guidelines. J Am Coll Cardiol 74, e177–e232. 30894318 10.1016/j.jacc.2019.03.010 PMC7685565

[3] Grundy SM , Bilheimer D , Blackburn H , et al. (1982) Rationale of the diet-heart statement of the American Heart Association. Report of Nutrition Committee. Circulation 65, 839A–854A. 7060268

[4] Mensink RP (2016) Effects of Saturated Fatty Acids on Serum Lipids and Lipoproteins: A Systematic Review and Regression Analysis. Geneve: WHO.

[5] Blomhoff R , Andersen R , Arnesen EK , et al. (2023) Nordic Nutrition Recommendations 2023. Copenhagen: Nordic Council of Ministers.

[6] Astrup A , Teicholz N , Magkos F , et al. (2021) Dietary saturated fats and health: are the U.S. guidelines evidence-based? Nutrients 13, 3305. 34684304 10.3390/nu13103305 PMC8541481

[7] Wang DD & Hu FB (2017) Dietary fat and risk of cardiovascular disease: recent controversies and advances. Annu Rev Nutr 37, 423–446. 28645222 10.1146/annurev-nutr-071816-064614

[8] Mozaffarian D , Micha R & Wallace S (2010) Effects on coronary heart disease of increasing polyunsaturated fat in place of saturated fat: a systematic review and meta-analysis of randomized controlled trials. PLoS Med 7, e1000252. 20351774 10.1371/journal.pmed.1000252 PMC2843598

[9] Sacks FM , Lichtenstein AH , Wu JHY , et al. (2017) Dietary fats and cardiovascular disease: a presidential advisory from the American Heart Association. Circulation 136, e1–e23. 28620111 10.1161/CIR.0000000000000510

[10] Hooper L , Martin N , Jimoh OF , et al. (2020) Reduction in saturated fat intake for cardiovascular disease. Cochrane Database Syst Rev 2020 issue 8, CD011737. 10.1002/14651858.CD011737.pub3 PMC8092457 32827219

[11] Siri-Tarino PW , Sun Q , Hu FB , et al. (2010) Meta-analysis of prospective cohort studies evaluating the association of saturated fat with cardiovascular disease. Am J Clin Nutr 91, 535–546. 20071648 10.3945/ajcn.2009.27725 PMC2824152

[12] Chowdhury R , Warnakula S , Kunutsor S , et al. (2014) Association of dietary, circulating, and supplement fatty acids with coronary risk: a systematic review and meta-analysis. Ann Intern Med 160, 398–406. 24723079 10.7326/M13-1788

[13] de Souza RJ , Mente A , Maroleanu A , et al. (2015) Intake of saturated and trans unsaturated fatty acids and risk of all cause mortality, cardiovascular disease, and type 2 diabetes: systematic review and meta-analysis of observational studies. BMJ 351, h3978. 10.1136/bmj.h3978 PMC4532752 26268692

[14] Mazidi M , Mikhailidis DP , Sattar N , et al. (2020) Association of types of dietary fats and all-cause and cause-specific mortality: a prospective cohort study and meta-analysis of prospective studies with 1 164 029 participants. Clin Nutr 39, 3677–3686. 32307197 10.1016/j.clnu.2020.03.028

[15] Reynolds AN , Hodson L , de Souza RJ , et al. (2022) Saturated Fat and Trans-Fat Intakes and their Replacement with Other Macronutrients: A Systematic Review and Meta-Analysis of Prospective Observational Studies. Geneva: World Health Organization.

[16] Astrup A , Dyerberg J , Elwood P , et al. (2011) The role of reducing intakes of saturated fat in the prevention of cardiovascular disease: where does the evidence stand in 2010? Am J Clin Nutr 93, 684–688. 21270379 10.3945/ajcn.110.004622 PMC3138219

[17] Kromhout D , Geleijnse JM , Menotti A , et al. (2011) The confusion about dietary fatty acids recommendations for CHD prevention. Br J Nutr 106, 627–632. 21733329 10.1017/S0007114511002236

[18] Barnard ND , Willett WC & Ding EL (2017) The misuse of meta-analysis in nutrition research. JAMA 318, 1435–1436. 28975260 10.1001/jama.2017.12083

[19] Hutcheon JA , Chiolero A & Hanley JA (2010) Random measurement error and regression dilution bias. BMJ 340, c2289. 10.1136/bmj.c2289 20573762

[20] Willett W (2013) Nutritional Epidemiology, 3rd ed. Oxford: Oxford University Press.

[21] Li Y , Hruby A , Bernstein AM , et al. (2015) Saturated fats compared with unsaturated fats and sources of carbohydrates in relation to risk of coronary heart disease: a prospective cohort study. J Am Coll Cardiol 66, 1538–1548. 26429077 10.1016/j.jacc.2015.07.055 PMC4593072

[22] Sellem L , Flourakis M , Jackson KG , et al. (2022) Impact of replacement of individual dietary SFAs on circulating lipids and other biomarkers of cardiometabolic health: a systematic review and meta-analysis of randomized controlled trials in humans. Adv Nutr 13, 1200–1225. 34849532 10.1093/advances/nmab143 PMC9340975

[23] Imamura F , Micha R , Wu JH , et al. (2016) Effects of saturated fat, polyunsaturated fat, monounsaturated fat, and carbohydrate on glucose-insulin homeostasis: a systematic review and meta-analysis of randomised controlled feeding trials. PLoS Med 13, e1002087. 27434027 10.1371/journal.pmed.1002087 PMC4951141

[24] Zong G , Li Y , Wanders AJ , et al. (2016) Intake of individual saturated fatty acids and risk of coronary heart disease in US men and women: two prospective longitudinal cohort studies. BMJ 355, i5796. 10.1136/bmj.i5796 PMC5121105 27881409

[25] Praagman J , Beulens JW , Alssema M , et al. (2016) The association between dietary saturated fatty acids and ischemic heart disease depends on the type and source of fatty acid in the European Prospective Investigation into Cancer and Nutrition-Netherlands cohort. Am J Clin Nutr 103, 356–365. 26791181 10.3945/ajcn.115.122671

[26] Praagman J , de Jonge EA , Kiefte-de Jong JC , et al. (2016) Dietary saturated fatty acids and coronary heart disease risk in a Dutch middle-aged and elderly population. Arterioscler Thromb Vasc Biol 36, 2011–2018. 27417581 10.1161/ATVBAHA.116.307578

[27] Praagman J , Vissers LET , Mulligan AA , et al. (2019) Consumption of individual saturated fatty acids and the risk of myocardial infarction in a UK and a Danish cohort. Int J Cardiol 279, 18–26. 30482628 10.1016/j.ijcard.2018.10.064 PMC6774776

[28] Zhuang P , Cheng L , Wang J , et al. (2019) Saturated fatty acid intake is associated with total mortality in a nationwide cohort study. J Nutr 149, 68–77. 30608597 10.1093/jn/nxy237

[29] Kromhout D , Menotti A , Bloemberg B , et al. (1995) Dietary saturated and trans fatty acids and cholesterol and 25-year mortality from coronary heart disease: the Seven Countries Study. Prev Med 24, 308–315. 7644455 10.1006/pmed.1995.1049

[30] Kabagambe EK , Baylin A , Siles X , et al. (2003) Individual saturated fatty acids and nonfatal acute myocardial infarction in Costa Rica. Eur J Clin Nutr 57, 1447–1457. 14576758 10.1038/sj.ejcn.1601709

[31] SACN (2019) Saturated Fats and Health. London: Public Health England.

[32] Snetselaar L , Bailey R , Sabate J , et al. (2020) Types of Dietary Fat and Cardiovascular Disease: A Systematic Review. Alexandria, VA: American Society for Nutrition. 35436064

[33] World Health Organization (2023) Saturated Fatty Acid and Trans-Fatty Acid Intake for Adults and Children: WHO Guideline. Geneva: World Health Organization. 37490572

[34] Pedersen JI , Tverdal A & Kirkhus B (2004) Diet changes and the rise and fall of cardiovascular disease mortality in Norway. J Norw Med Assoc 124, 1532–1536. 15195160

[35] Johansson L , Drevon CA & Aa Bjorneboe GE (1996) The Norwegian diet during the last hundred years in relation to coronary heart disease. Eur J Clin Nutr 50, 277–283. 8735307

[36] Laake I , Pedersen JI , Selmer R , et al. (2012) A prospective study of intake of trans-fatty acids from ruminant fat, partially hydrogenated vegetable oils, and marine oils and mortality from CVD. Br J Nutr 108, 743–754. 22059639 10.1017/S0007114511005897

[37] Bjartveit K , Foss OP , Gjervig T , et al. (1979) The cardiovascular disease study in Norwegian counties. Background and organization. Acta Med Scand Suppl 634, 1–70. 293122

[38] Bjartveit K , Foss OP & Gjervig T (1983) The cardiovascular disease study in Norwegian counties. Results from first screening. Acta Med Scand Suppl 675, 1–184. 6581684

[39] Lindman AS , Veierod MB , Tverdal A , et al. (2010) Nonfasting triglycerides and risk of cardiovascular death in men and women from the Norwegian Counties Study. Eur J Epidemiol 25, 789–798. 20890636 10.1007/s10654-010-9501-1 PMC2991549

[40] Løken EB & Solvoll K (1997) Can dietary data from the cardiovascular disease studies in Finnmark, Sogn og Fjordane and Oppland be used to analyse risk for other diseases? Nor J Epidemiol 7, 191–200.

[41] Gaard M , Tretli S & Loken EB (1995) Dietary fat and the risk of breast cancer: a prospective study of 25 892 Norwegian women. Int J Cancer 63, 13–17. 7558440 10.1002/ijc.2910630104

[42] Blaker B & Aarsland M (1995) Measures and *Weights of Foods*, 2nd ed. Oslo: Landsforeningen for kosthold & helse.

[43] Blaker B & Rimestad AH (1991) National Nutrition Council’s Food Composition Table, 6th ed. Oslo: Landsforeningen for kosthold og helse.

[44] Solvoll K (1983) Comparison of Dietary Data from Self-Administered Questionnaire and 24 *hour* Recall [In Norwegian], The Cardiovascular Disease Study in Norwegian Counties. Oslo: Section for Dietary Research, University of Oslo.

[45] Solvoll K , Løken EB , Grønn M , et al. (1985) Dietary Data from the Municipality of Vestre Toten 1982 : Results from 24 h Recall among Men and Women 25–54 Years Old [In Norwegian]. Oslo: Section for Dietary Research, University of Oslo.

[46] Blaker B , Solvoll K & Lund-Larsen K (1988) Dietary Data from the Municipality of Vestre Toten 1987: Results from 24 h Recall among Men and Women 30–59 Years Old [In Norwegian]. Oslo: Section for Dietary Research, University of Oslo.

[47] Grimby G , Börjesson M , Jonsdottir IH , et al. (2015) The “Saltin–Grimby Physical Activity Level Scale” and its application to health research. Scand J Med Sci Sports 25, 119–125. 26589125 10.1111/sms.12611

[48] Foss OP & Urdal P (2003) Cholesterol through more than 25 years: can the results be compared over so long time? Nor J Epidemiol 13, 85–88.

[49] Nordestgaard BG , Langlois MR , Langsted A , et al. (2020) Quantifying atherogenic lipoproteins for lipid-lowering strategies: consensus-based recommendations from EAS and EFLM. Atherosclerosis 294, 46–61. 31928713 10.1016/j.atherosclerosis.2019.12.005

[50] Willett WC , Howe GR & Kushi LH (1997) Adjustment for total energy intake in epidemiologic studies. Am J Clin Nutr 65, 1220S–1228S; discussion 1229S-1231S. 9094926 10.1093/ajcn/65.4.1220S

[51] Hu FB , Stampfer MJ , Rimm E , et al. (1999) Dietary fat and coronary heart disease: a comparison of approaches for adjusting for total energy intake and modeling repeated dietary measurements. Am J Epidemiol 149, 531–540. 10084242 10.1093/oxfordjournals.aje.a009849

[52] Wang DD , Li Y , Chiuve SE , et al. (2016) Association of specific dietary fats with total and cause-specific mortality. JAMA Intern Med 176, 1134–1145. 27379574 10.1001/jamainternmed.2016.2417 PMC5123772

[53] Aune D , Keum N , Giovannucci E , et al. (2018) Dietary intake and blood concentrations of antioxidants and the risk of cardiovascular disease, total cancer, and all-cause mortality: a systematic review and dose-response meta-analysis of prospective studies. Am J Clin Nutr 108, 1069–1091. 30475962 10.1093/ajcn/nqy097 PMC6250988

[54] Ma Y , He FJ , Sun Q , et al. (2022) 24-hour urinary sodium and potassium excretion and cardiovascular risk. N Engl J Med 386, 252–263. 34767706 10.1056/NEJMoa2109794 PMC9153854

[55] Jakobsen MU , O’Reilly EJ , Heitmann BL , et al. (2009) Major types of dietary fat and risk of coronary heart disease: a pooled analysis of 11 cohort studies. Am J Clin Nutr 89, 1425–1432. 19211817 10.3945/ajcn.2008.27124 PMC2676998

[56] Farvid MS , Ding M , Pan A , et al. (2014) Dietary linoleic acid and risk of coronary heart disease: a systematic review and meta-analysis of prospective cohort studies. Circulation 130, 1568–1578. 25161045 10.1161/CIRCULATIONAHA.114.010236 PMC4334131

[57] Øgrim ME , Solvang A , Berge S , et al. (1981) Norwegian Diet 1975–79: A Study of Food Supplies and Foods Entering the Household as Sources of Information about the Composition and Changes in the Norwegian Diet 1975–1979. Oslo: Section for dietary research, University of Oslo.

[58] Jacobs DR Jr , Meyer HE & Solvoll K (2001) Reduced mortality among whole grain bread eaters in men and women in the Norwegian County Study. Eur J Clin Nutr 55, 137–143. 11305627 10.1038/sj.ejcn.1601133

[59] Reynolds A , Mann J , Cummings J , et al. (2019) Carbohydrate quality and human health: a series of systematic reviews and meta-analyses. Lancet 393, 434–445. 30638909 10.1016/S0140-6736(18)31809-9

[60] Zhuang P , Zhang Y , He W , et al. (2019) Dietary fats in relation to total and cause-specific mortality in a prospective cohort of 521 120 individuals with 16 years of follow-up. Circ Res 124, 757–768. 30636521 10.1161/CIRCRESAHA.118.314038

[61] Pedersen JI , Ringstad J , Almendingen K , et al. (2000) Adipose tissue fatty acids and risk of myocardial infarction--a case-control study. Eur J Clin Nutr 54, 618–625. 10951510 10.1038/sj.ejcn.1601064

[62] Wu J , Wilson KM , Stampfer MJ , et al. (2018) A 24-year prospective study of dietaryα-linolenic acid and lethal prostate cancer. Int J Cancer 142, 2207–2214. 29315549 10.1002/ijc.31247 PMC5893397

[63] Zong G , Li Y , Sampson L , et al. (2018) Monounsaturated fats from plant and animal sources in relation to risk of coronary heart disease among US men and women. Am J Clin Nutr 107, 445–453. 29566185 10.1093/ajcn/nqx004 PMC5875103

[64] Steur M , Johnson L , Sharp SJ , et al. (2021) Dietary fatty acids, macronutrient substitutions, food sources and incidence of coronary heart disease: findings from the EPIC-CVD Case-Cohort Study across nine European countries. J Am Heart Assoc 10, e019814. 34796724 10.1161/JAHA.120.019814 PMC9075396

[65] Haugsgjerd TR , Egeland GM , Nygard OK , et al. (2022) Intake of carbohydrates and SFA and risk of CHD in middle-age adults: the Hordaland Health Study (HUSK). Public Health Nutr 25, 634–648. 32907659 10.1017/S1368980020003043 PMC9991815

[66] Chen M , Li Y , Sun Q , et al. (2016) Dairy fat and risk of cardiovascular disease in 3 cohorts of US adults. Am J Clin Nutr 104, 1209–1217. 27557656 10.3945/ajcn.116.134460 PMC5081717

[67] Hu FB , Stampfer MJ , Manson JE , et al. (1999) Dietary saturated fats and their food sources in relation to the risk of coronary heart disease in women. Am J Clin Nutr 70, 1001–1008. 10584044 10.1093/ajcn/70.6.1001

[68] Watts GF , Jackson P , Burke V , et al. (1996) Dietary fatty acids and progression of coronary artery disease in men. Am J Clin Nutr 64, 202–209. 8694021 10.1093/ajcn/64.2.202

[69] Micha R & Mozaffarian D (2010) Saturated fat and cardiometabolic risk factors, coronary heart disease, stroke, and diabetes: a fresh look at the evidence. Lipids 45, 893–905. 20354806 10.1007/s11745-010-3393-4 PMC2950931

[70] Ference BA , Graham I , Tokgozoglu L , et al. (2018) Impact of lipids on cardiovascular health: JACC health promotion series. J Am Coll Cardiol 72, 1141–1156. 30165986 10.1016/j.jacc.2018.06.046

[71] Boniface DR & Tefft ME (2002) Dietary fats and 16-year coronary heart disease mortality in a cohort of men and women in Great Britain. Eur J Clin Nutr 56, 786–792. 12122556 10.1038/sj.ejcn.1601509

[72] Jakobsen MU , Overvad K , Dyerberg J , et al. (2004) Dietary fat and risk of coronary heart disease: possible effect modification by gender and age. Am J Epidemiol 160, 141–149. 15234935 10.1093/aje/kwh193

[73] Nagata C , Nakamura K , Wada K , et al. (2012) Total fat intake is associated with decreased mortality in Japanese men but not in women. J Nutr 142, 1713–1719. 22810986 10.3945/jn.112.161661

[74] Liu X , Harding SV & Rideout TC (2022) Saturated fat and cardiovascular health: phenotype and dietary factors influencing interindividual responsiveness. Curr Atheroscler Rep 24, 391–398. 35320834 10.1007/s11883-022-01014-w

[75] Flock MR , Green MH & Kris-Etherton PM (2011) Effects of adiposity on plasma lipid response to reductions in dietary saturated fatty acids and cholesterol. Adv Nutr 2, 261–274. 22332058 10.3945/an.111.000422 PMC3090171

[76] Sundfør TM , Svendsen M , Heggen E , et al. (2019) BMI modifies the effect of dietary fat on atherogenic lipids: a randomized clinical trial. Am J Clin Nutr 110, 832–841. 31216575 10.1093/ajcn/nqz113

[77] Petersen KS , Bowen KJ , Tindall AM , et al. (2020) The effect of inflammation and insulin resistance on lipid and lipoprotein responsiveness to dietary intervention. Curr Dev Nutr 4, nzaa160. 10.1093/cdn/nzaa160 PMC7792751 33447695

[78] Keogh RH , Shaw PA , Gustafson P , et al. (2020) STRATOS guidance document on measurement error and misclassification of variables in observational epidemiology: part 1-Basic theory and simple methods of adjustment. Stat Med 39, 2197–2231. 32246539 10.1002/sim.8532 PMC7450672

[79] Laake I , Thune I , Selmer R , et al. (2010) A prospective study of body mass index, weight change, and risk of cancer in the proximal and distal colon. Cancer Epidemiol Biomarkers Prev 19, 1511–1522. 20501754 10.1158/1055-9965.EPI-09-0813

[80] Ding M , Li J , Qi L , et al. (2019) Associations of dairy intake with risk of mortality in women and men: three prospective cohort studies. BMJ 367, l6204. 10.1136/bmj.l6204 PMC6880246 31776125

[81] Jakobsen MU , Trolle E , Outzen M , et al. (2021) Intake of dairy products and associations with major atherosclerotic cardiovascular diseases: a systematic review and meta-analysis of cohort studies. Sci Rep 11, 1303. 33446728 10.1038/s41598-020-79708-x PMC7809206

[82] Van Parys A , Saele J , Puaschitz NG , et al. (2023) The association between dairy intake and risk of cardiovascular disease and mortality in patients with stable angina pectoris. Eur J Prev Cardiol 30, 219–229. 36134600 10.1093/eurjpc/zwac217

[83] Brayner B , Kaur G , Keske MA , et al. (2021) Dietary patterns characterized by fat type in association with obesity and type 2 diabetes: a longitudinal study of UK Biobank participants. J Nutr 151, 3570–3578. 34522964 10.1093/jn/nxab275

[84] Rhee JJ , Cho E & Willett WC (2014) Energy adjustment of nutrient intakes is preferable to adjustment using body weight and physical activity in epidemiological analyses. Public Health Nutr 17, 1054–1060. 23701939 10.1017/S1368980013001390 PMC3884063

[85] Pedersen AG & Ellingsen CL (2015) Data quality in the causes of death registry. Tidsskr Nor Laegeforen 135, 768–770. 25947599 10.4045/tidsskr.14.1065

[86] Gulsvik AK , Gulsvik A , Svendsen E , et al. (2011) Diagnostic validity of fatal cerebral strokes and coronary deaths in mortality statistics: an autopsy study. Eur J Epidemiol 26, 221–228. 21170572 10.1007/s10654-010-9535-4 PMC3079075

