# Supplementary material for: Saturated fatty acids and total and CVD mortality in Norway: a prospective cohort study with up to 45 years of follow-up
Source: Br J Nutr. 2024 Sep 16;132(4):466–78. doi: 10.1017/S0007114524001351 (PMC11499087; doi:10.1017/S0007114524001351)
Supplement: Arnesen et al. supplementary material [file S0007114524001351sup001.docx]

**Saturated fatty acids and total and cardiovascular disease mortality in Norway: A prospective cohort study with up to 45 years of follow-up (Arnesen et al.)**

**Supplementary material**

# Supplementary Table 1. Number of residents invited to and attending each screening.

| County | Time for screening | N invited | N attending | % attendance |
| --- | --- | --- | --- | --- |
| **Screening I** | | | | |
| Finnmark | 1974-75 | 17,401 | 14,340 | 82.4 |
| Sogn og Fjordane | 1975-76 | 16,603 | 14,966 | 90.1 |
| Oppland | 1976-78 | 31,620 | 28,399 | 89.8 |
| *Total* | | *65,624* | *57,705* | *87.9* |
| **Screening II** | | | | |
| Finnmark | 1977-78 | 20,647 | 17,145 | 83.0 |
| Sogn og Fjordane | 1980-81 | 19,506 | 17,473 | 89.6 |
| Oppland | 1981-83 | 31,581 | 28,437 | 89.8 |
| *Total* | | *71,734* | *63,055* | *87.5* |
| **Screening III** | | | | |
| Finnmark | 1987-88 | 22,994 | 17,852 | 77.6 |
| Sogn og Fjordane | 1985-86 | 21,423 | 18,669 | 87.1 |
| Oppland | 1986-88 | 37,270 | 32,124 | 86.2 |
| *Total* | | *81,687* | *68,645* | *83.6* |


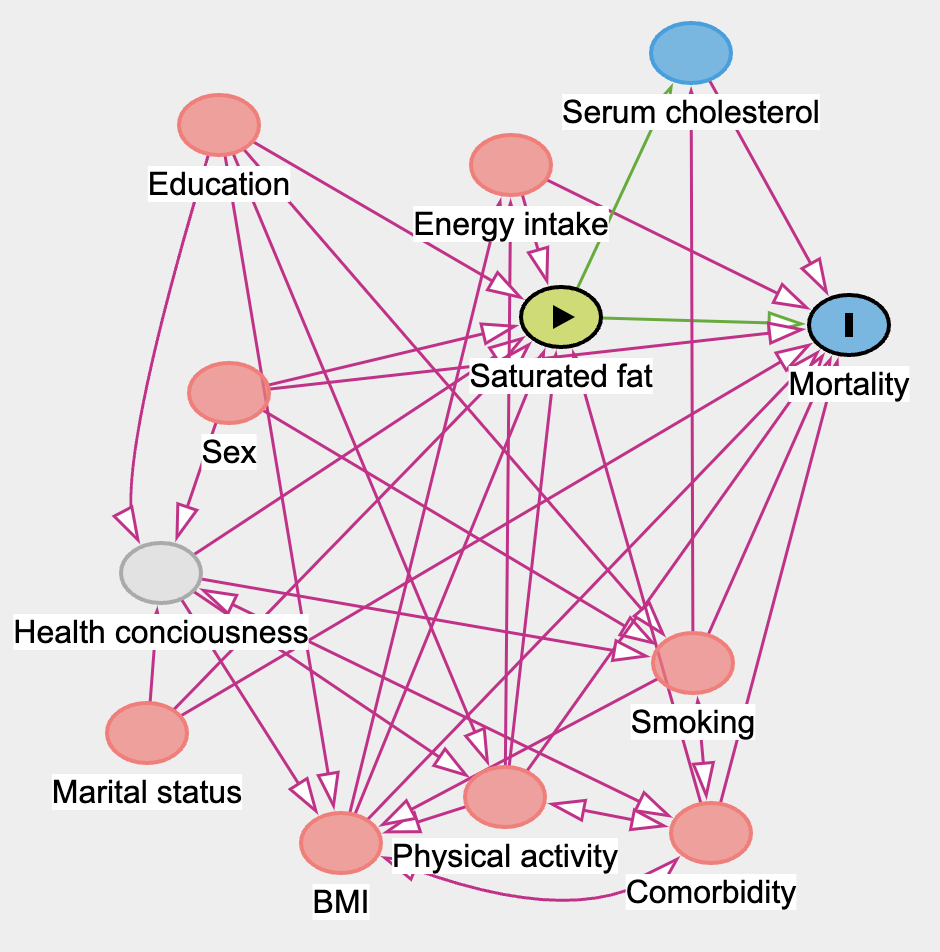


# Supplementary Figure 1. Directed acyclic graph of the hypothesised association between intake of saturated fats (exposure, green node) and total/cardiovascular disease mortality (outcome, blue node). The green arrows represent the causal relationship. The red nodes are ancestors of both the exposure and outcome. The grey node represents the unmeasured variable (health consciousness). The graph suggests that the minimal sufficient adjustment to estimate the total effect of saturated fat includes body mass index (BMI), energy intake, sex, physical activity, comorbidity, smoking, and marital status.

# Supplementary Table 2. Baseline characteristics of the included sample and persons excluded due to lack of dietary data.

|  | Study sample  (n = 78,725) | Without  (n = 11,847) |
| --- | --- | --- |
| Male, % | 50.4 | 51.8 |
| Age, years, mean (SD) | 41.1 (7.2) | 39.1 (7.9) |
| Higher education (%) | 10.1 | 9.9 |
| BMI, kg/m^2^ | 24.8 (3.6) | 24.6 (3.7) |
| BMI ≥ 30 kg/m^2^ (%) | 8.0 | 8.0 |
| Total cholesterol, mean (SD) mmol/l | 6.12 (1.23) | 6.16 (1.31) |
| Systolic blood pressure, mean (SD) mmHg | 133 (17) | 132 (17) |
| Current smoking, % | 44.1 | 54.6 |
| Inactive leisure, % | 18.8 | 23.5 |
| Sedentary work, % | 22.4 | 22.9 |
| Married, % | 80.9 | 71.5 |
| History of MI, % | 0.5 | 0.6 |
| History of stroke, % | 0.2 | 0.3 |
| History of diabetes, % | 0.7 | 0.8 |

# Supplementary Figure 2. Mean total (left) and non-HDL cholesterol (right) levels by quintile of E% from SFA, estimated from linear mixed-effects regression accounting for intra-individual correlations. Adjusted for age, sex, comorbidities, education, BMI, smoking status, physical activity, energy intake, MUFA, PUFA, trans-FA, protein, and dietary cholesterol. In this model, the comparator is carbohydrates. N = 78,690 for total cholesterol, 66,717 for non-HDL-cholesterol.

# Supplementary Table 3. Hazard ratios (95 % confidence intervals) of total, CVD, IHD, and AMI mortality by quintile and per 5 E% intake of saturated fat, stratified by sex (n = 78,725).

|  | | Hazard ratios (95 % CI)^1^ | | | | | | | | | | | | | | | | | |
| --- | --- | --- | --- | --- | --- | --- | --- | --- | --- | --- | --- | --- | --- | --- | --- | --- | --- | --- | --- |
|  | | Q1 | | Q2 | | Q3 | | Q4 | | Q5 | | *p*_interaction_^2^ | | Per 5 E% | | *p*_trend_ | | *p*_interaction_^2^ | |
| ***Total mortality*** | | | | | | | | | | | | 0.641 | |  | |  | | 0.140 | |
| Males | |  | |  | |  | |  | |  | |  | |  | |  | |  | |
| Deaths, n | | 3492 | | 3393 | | 3328 | | 3205 | | 3289 | |  | |  | |  | |  | |
| HR (95% CI) | | 1 (ref) | | 1.03 (0.98, 1.08) | | 1.04 (0.98, 1.10) | | 1.10 (1.03, 1.17) | | 1.20 (1.10, 1.30) | |  | | 1.16 (1.10, 1.23) | | <0.001 | |  | |
| Females | |  | |  | |  | |  | |  | |  | |  | |  | |  | |
| Deaths, n | | 2479 | | 2618 | | 2646 | | 2305 | | 1800 | |  | |  | |  | |  | |
| HR (95% CI) | | 1 (ref) | | 1.03 (0.97, 1.10) | | 1.07 (1.00, 1.14) | | 1.12 (1.03, 1.21) | | 1.27 (1.15, 1.40) | |  | | 1.15 (1.08, 1.24) | | <0.001 | |  | |
| ***CVD mortality*** | | | | | | | | | | | | 0.015 | |  | |  | | <0.001 | |
| Males | |  | |  | |  | |  | |  | |  | |  | |  | |  | |
| Deaths, n | | 1337 | | 1211 | | 1167 | | 1125 | | 1134 | |  | |  | |  | |  | |
| HR (95% CI) | | 1 (ref) | | 0.99 (0.91, 1.08) | | 1.00 (0.91, 1.10) | | 1.06 (0.95, 1.18) | | 1.15 (1.00, 1.31) | |  | | 1.11 (1.01, 1.22) | | 0.034 | |  | |
| Females | |  | |  | |  | |  | |  | |  | |  | |  | |  | |
| Deaths, n | | 682 | | 751 | | 750 | | 629 | | 532 | |  | |  | |  | |  | |
| HR (95% CI) | | 1 (ref) | | 1.07 (0.95, 1.20) | | 1.07 (0.95, 1.22) | | 1.10 (0.95, 1.28) | | 1.35 (1.12, 1.62) | |  | | 1.22 (1.07, 1.38) | | 0.002 | |  | |
| ***IHD mortality*** | | | | | | | | | | | | 0.001 | |  | |  | | <0.001 | |
| Males | |  | |  | |  | |  | |  | |  | |  | |  | |  | |
| Deaths, n | | 771 | | 666 | | 659 | | 640 | | 659 | |  | |  | |  | |  | |
| HR (95% CI) | | 1 (ref) | | 0.98 (0.88, 1.10) | | 1.02 (0.90, 1.16) | | 1.09 (0.94, 1.25) | | 1.22 (1.02, 1.47) | |  | | 1.11 (0.98, 1.25) | | 0.107 | |  | |
| Females | |  | |  | |  | |  | |  | |  | |  | |  | |  | |
| Deaths, n | | 234 | | 301 | | 316 | | 272 | | 213 | |  | |  | |  | |  | |
| HR (95% CI) | | 1 (ref) | | 1.24 (1.03, 1.50) | | 1.30 (1.06, 1.60) | | 1.38 (1.08, 1.75) | | 1.53 (1.14, 2.07) | |  | | 1.25 (1.02, 1.52) | | 0.029 | |  | |
| ***AMI mortality*** | | | | | | | | | | | | 0.005 | |  | |  | | 0.002 | |
| Males | |  | |  | |  | |  | |  | |  | |  | |  | |  | |
| Deaths, n | | 538 | | 452 | | 434 | | 443 | | 455 | |  | |  | |  | |  | |
| HR (95% CI) | | 1 (ref) | | 0.96 (0.84, 1.10) | | 0.97 (0.84, 1.13) | | 1.08 (0.91, 1.28) | | 1.22 (0.98, 1.51) | |  | | 1.15 (0.99, 1.34) | | 0.067 | |  | |
| Females | |  | |  | |  | |  | |  | |  | |  | |  | |  | |
| Deaths, n | | 172 | | 218 | | 214 | | 196 | | 160 | |  | |  | |  | |  | |
| HR (95% CI) | | 1 (ref) | | 1.24 (1.00, 1.55) | | 1.24 (0.97, 1.58) | | 1.41 (1.06, 1.86) | | 1.67 (1.18, 2.37) | |  | | 1.22 (0.96, 1.54) | | 0.097 | |  | |

AMI: Acute myocardial infarction. CVD: cardiovascular disease. IHD: ischaemic heart disease. SFA: saturated fatty acids. ^1^ Cox proportional hazards regression stratified by birth cohort (<1930, 1930-34, 1935-39, 1940-44, 1945-49, 1950-54, and ≥1955) with age as timescale, adjusted for energy intake, body mass index, physical activity, smoking habits, attained education, marital status, self-reported comorbidities (i.e., history of myocardial infarction, stroke, angina or diabetes, treatment for high blood pressure or use of nitro-glycerine), and energy from other types of fat and protein (in E%) and cholesterol (in mg/1000 kcal); the HRs in this model can be interpreted as the associations with intakes of SFA at the expense of carbohydrates.
^2^*p* value for multiplicative interaction calculated from the likelihood ratio test comparing the model with and without the products terms.

# Supplementary Table 4. Hazard ratios (95 % confidence intervals) of total, CVD, IHD, and AMI mortality by quintile and per 5 E% intake of saturated fat, stratified by BMI category (n = 78,725).

|  | Hazard ratios (95 % CI)^1^ | | | | | | | | |
| --- | --- | --- | --- | --- | --- | --- | --- | --- | --- |
|  | Q1 | Q2 | Q3 | Q4 | Q5 | *p*_interaction_^2^ | Per 5 E% | *p*_trend_ | *p*_interaction_^2^ |
| ***Total mortality*** | | | | | | 0.004 |  |  | 0.003 |
| <18.5 |  |  |  |  |  |  |  |  |  |
| Deaths, n | 48 | 57 | 87 | 87 | 78 |  |  |  |  |
| HR (95% CI) | 1 (ref) | 0.94 (0.62, 1.43) | 1.18 (0.76, 1.84) | 1.19 (0.73, 1.94) | 0.75 (0.41, 1.37) |  | 0.71 (0.48, 1.03) | 0.073 |  |
| 18.5-24.9 |  |  |  |  |  |  |  |  |  |
| Deaths, n | 2280 | 2554 | 2790 | 2671 | 2640 |  |  |  |  |
| HR (95% CI) | 1 (ref) | 1.03 (0.97, 1.10) | 1.06 (0.99, 1.14) | 1.14 (1.06, 1.23) | 1.27 (1.15, 1.39) |  | 1.21 (1.13, 1.28) | <0.001 |  |
| 25.0-29.9 |  |  |  |  |  |  |  |  |  |
| Deaths, n | 2654 | 2488 | 2329 | 2216 | 1892 |  |  |  |  |
| HR (95% CI) | 1 (ref) | 1.04 (0.98, 1.10) | 1.08 (1.00, 1.15) | 1.16 (1.07, 1.25) | 1.30 (1.18, 1.43) |  | 1.20 (1.12, 1.28) | <0.001 |  |
| ≥30 |  |  |  |  |  |  |  |  |  |
| Deaths, n | 989 | 912 | 768 | 536 | 479 |  |  |  |  |
| HR (95% CI) | 1 (ref) | 1.06 (0.96, 1.17) | 1.01 (0.90, 1.13) | 0.95 (0.83, 1.09) | 1.14 (0.96, 1.36) |  | 1.05 (0.94, 1.18) | 0.375 |  |
| ***CVD mortality*** | | | | | | 0.278 |  |  | 0.051 |
| <18.5 |  |  |  |  |  |  |  |  |  |
| Deaths, n | 15 | 7 | 16 | 18 | 16 |  |  |  |  |
| HR (95% CI) | 1 (ref) | 0.40 (0.15, 1.11) | 0.81 (0.31, 2.10) | 0.96 (0.35, 2.65) | 0.57 (0.15, 2.12) |  | 0.69 (0.30, 1.56) | 0.373 |  |
| 18.5-24.9 |  |  |  |  |  |  |  |  |  |
| Deaths, n | 641 | 721 | 797 | 751 | 772 |  |  |  |  |
| HR (95% CI) | 1 (ref) | 1.05 (0.94, 1.18) | 1.09 (0.96, 1.23) | 1.15 (1.00, 1.32) | 1.32 (1.11, 1.57) |  | 1.25 (1.11, 1.40) | <0.001 |  |
| 25.0-29.9 |  |  |  |  |  |  |  |  |  |
| Deaths, n | 959 | 875 | 804 | 780 | 693 |  |  |  |  |
| HR (95% CI) | 1 (ref) | 1.00 (0.91, 1.11) | 1.02 (0.91, 1.14) | 1.09 (0.96, 1.24) | 1.21 (1.03, 1.43) |  | 1.12 (1.00, 1.25) | 0.052 |  |
| ≥30 |  |  |  |  |  |  |  |  |  |
| Deaths, n | 404 | 359 | 300 | 205 | 185 |  |  |  |  |
| HR (95% CI) | 1 (ref) | 1.09 (0.93, 1.28) | 1.03 (0.86, 1.24) | 0.95 (0.76, 1.18) | 1.19 (0.90, 1.57) |  | 1.07 (0.89, 1.28) |  |  |
| ***IHD mortality*** | | | | | | 0.733 |  |  | 0.988 |
| <18.5 |  |  |  |  |  |  |  |  |  |
| Deaths, n | 7 | 3 | 11 | 5 | 4 |  |  |  |  |
| HR (95% CI) | 1 (ref) | 0.36 (0.08, 1.67) | 1.04 (0.26, 4.13) | 0.42 (0.08, 2.31) | 0.16 (0.01, 66) |  | 0.43 (0.12, 1.62) | 0.212 |  |
| 18.5-24.9 |  |  |  |  |  |  |  |  |  |
| Deaths, n | 325 | 341 | 376 | 377 | 390 |  |  |  |  |
| HR (95% CI) | 1 (ref) | 1.04 (0.88, 1.22) | 1.09 (0.91, 1.31) | 1.24 (1.01, 1.51) | 1.47 (1.15, 1.88) |  | 1.33 (1.12, 1.57) | 0.001 |  |
| 25.0-29.9 |  |  |  |  |  |  |  |  |  |
| Deaths, n | 482 | 441 | 433 | 419 | 375 |  |  |  |  |
| HR (95% CI) | 1 (ref) | 1.04 (0.90, 1.20) | 1.14 (0.97, 1.33) | 1.19 (0.99, 1.42) | 1.29 (1.03, 1.62) |  | 1.10 (0.94, 1.28) | 0.233 |  |
| ≥30 |  |  |  |  |  |  |  |  |  |
| Deaths, n | 191 | 182 | 155 | 111 | 103 |  |  |  |  |
| HR (95% CI) | 1 (ref) | 1.13 (0.90, 1.41) | 1.04 (0.81, 1.35) | 0.95 (0.70, 1.30) | 1.12 (0.76, 1.65) |  | 0.96 (0.74, 1.24) | 0.735 |  |
| ***AMI mortality*** | | | | | | 0.748 |  |  | 0.804 |
| <18.5 |  |  |  |  |  |  |  |  |  |
| Deaths, n | 6 | 3 | 7 | 4 | 3 |  |  |  |  |
| HR (95% CI) | 1 (ref) | 0.46 (0.09, 2.36) | 0.81 (0.16, 4.01) | 0.41 (0.06, 2.91) | 0.15 (0.01, 2.20) |  | 0.46 (0.11, 2.00) | 0.301 |  |
| 18.5-24.9 |  |  |  |  |  |  |  |  |  |
| Deaths, n | 221 | 231 | 251 | 258 | 267 |  |  |  |  |
| HR (95% CI) | 1 (ref) | 1.03 (0.85, 1.26) | 1.07 (0.86, 1.34) | 1.24 (0.98, 1.58) | 1.45 (1.08, 1.97) |  | 1.33 (1.08, 1.63) | 0.007 |  |
| 25.0-29.9 |  |  |  |  |  |  |  |  |  |
| Deaths, n | 342 | 313 | 283 | 297 | 265 |  |  |  |  |
| HR (95% CI) | 1 (ref) | 1.04 (0.87, 1.23) | 1.04 (0.86, 1.26) | 1.19 (0.96, 1.47) | 1.32 (1.01, 1.73) |  | 1.10 (0.92, 1.33) | 0.301 |  |
| ≥30 |  |  |  |  |  |  |  |  |  |
| Deaths, n | 141 | 123 | 107 | 80 | 80 |  |  |  |  |
| HR (95% CI) | 1 (ref) | 1.04 (0.80, 1.36) | 0.98 (0.72, 1.33) | 0.94 (0.66, 1.36) | 1.20 (0.77, 1.88) |  | 1.01 (0.75, 1.37) | 0.931 |  |

AMI: Acute myocardial infarction. CVD: cardiovascular disease. IHD: ischaemic heart disease. SFA: saturated fatty acids. ^1^ Cox proportional hazards regression stratified by birth cohort (<1930, 1930-34, 1935-39, 1940-44, 1945-49, 1950-54, and ≥1955) with age as timescale, adjusted sex, energy intake, physical activity, smoking habits, attained education, marital status, self-reported comorbidities (i.e., history of myocardial infarction, stroke, angina or diabetes, treatment for high blood pressure or use of nitro-glycerine), and energy from other types of fat and protein (in E%) and cholesterol (in mg/1000 kcal); the HRs in this model can be interpreted as the associations with intakes of SFA at the expense of carbohydrates.
^2^*p* value for multiplicative interaction calculated from the likelihood ratio test comparing the model with and without the products terms.

# Supplementary Table 5. Hazard ratios (95 % confidence intervals) of total, CVD, IHD, and AMI mortality by quintile and per 5 E% intake of saturated fat, stratified by presence of comorbidities (n = 78,725).

|  | Hazard ratios (95 % CI)^1^ | | | | | | | | |
| --- | --- | --- | --- | --- | --- | --- | --- | --- | --- |
|  | Q1 | Q2 | Q3 | Q4 | Q5 | *p*_interaction_^2^ | Per 5 E% | *p*_trend_ | *p*_interaction_^2^ |
| ***Total mortality*** | | | | | | <0.001 |  |  | 0.095 |
| No |  |  |  |  |  |  |  |  |  |
| Deaths, n | 4689 | 4878 | 5003 | 4748 | 4505 |  |  |  |  |
| HR (95% CI) | 1 (ref) | 1.03 (0.99, 1.08) | 1.07 (1.02, 1.12) | 1.15 (1.08, 1.21) | 1.27 (1.19, 1.36) |  | 1.20 (1.14, 1.25) | <0.001 |  |
| Yes |  |  |  |  |  |  |  |  |  |
| Deaths, n | 1282 | 1133 | 971 | 762 | 584 |  |  |  |  |
| HR (95% CI) | 1 (ref) | 1.05 (0.96, 1.15) | 1.05 (0.94, 1.16) | 1.05 (0.93, 1.19) | 1.21 (1.03, 1.42) |  | 1.13 (1.01, 1.25) | 0.029 |  |
| ***CVD mortality*** | | | | | | <0.001 |  |  | 0.467 |
| No |  |  |  |  |  |  |  |  |  |
| Deaths, n | 1420 | 1439 | 1481 | 1390 | 1367 |  |  |  |  |
| HR (95% CI) | 1 (ref) | 1.01(0.94, 1.10) | 1.05 (0.96, 1.14) | 1.12 (1.01, 1.23) | 1.26 (1.11, 1.42) |  | 1.19 (1.09, 1.29) | <0.001 |  |
| Yes |  |  |  |  |  |  |  |  |  |
| Deaths, n | 599 | 523 | 436 | 364 | 299 |  |  |  |  |
| HR (95% CI) | 1 (ref) | 1.07 (0.94, 1.22) | 1.05 (0.90, 1.23) | 1.11 (0.93, 1.33) | 1.36 (1.08, 1.71) |  | 1.18 (1.01, 1.38) | 0.038 |  |
| ***IHD mortality*** | | | | | | 0.009 |  |  | 0.901 |
| No |  |  |  |  |  |  |  |  |  |
| Deaths, n | 647 | 665 | 729 | 686 | 691 |  |  |  |  |
| HR (95% CI) | 1 (ref) | 1.07 (0.95, 1.20) | 1.19 (1.04, 1.35) | 1.27 (1.10, 1.46) | 1.45 (1.22, 1.74) |  | 1.20 (1.07, 1.36) |  |  |
| Yes |  |  |  |  |  |  |  |  |  |
| Deaths, n | 358 | 302 | 246 | 226 | 181 |  |  |  |  |
| HR (95% CI) | 1 (ref) | 1.00 (0.85, 1.19) | 0.94 (0.77, 1.15) | 1.06 (0.84, 1.33) | 1.16 (0.86, 1.57) |  | 1.11 (0.90, 1.35) |  |  |
| ***AMI mortality*** | | | | | | 0.024 |  |  | 0.263 |
| No |  |  |  |  |  |  |  |  |  |
| Deaths, n | 466 | 478 | 488 | 490 | 484 |  |  |  |  |
| HR (95% CI) | 1 (ref) | 1.09 (0.95, 1.25) | 1.15 (0.98, 1.33) | 1.33 (1.12, 1.57) | 1.54 (1.24, 1.90) |  | 1.24 (1.07, 1.43) | 0.004 |  |
| Yes |  |  |  |  |  |  |  |  |  |
| Deaths, n | 244 | 192 | 160 | 149 | 131 |  |  |  |  |
| HR (95% CI) | 1 (ref) | 0.90 (0.73, 1.11) | 0.85 (0.66, 1.08) | 0.93 (0.71, 1.23) | 1.05 (0.74, 1.51) |  | 1.07 (0.83, 1.36) | 0.607 |  |

AMI: Acute myocardial infarction. CVD: cardiovascular disease. IHD: ischaemic heart disease. SFA: saturated fatty acids. ^1^ Cox proportional hazards regression models were stratified by birth cohort (<1930, 1930-34, 1935-39, 1940-44, 1945-49, 1950-54, and ≥1955) with age as timescale, adjusted for sex, energy intake, body mass index, physical activity, smoking habits, attained education, marital status, and energy from other types of fat and protein (in E%) and cholesterol (in mg/1000 kcal); the HRs in this model can be interpreted as the associations with intakes of SFA at the expense of carbohydrates.
^2^*p* value for multiplicative interaction calculated from the likelihood ratio test comparing the model with and without the products terms.

# Supplementary Table 6. Associations between theoretical isocaloric substitution of energy from saturated fats with other macronutrients and risk of total, CVD, IHD, and AMI mortality.

| Substitution | Total mortality | | CVD mortality | | IHD mortality | | AMI mortality | |
| --- | --- | --- | --- | --- | --- | --- | --- | --- |
|  | HR (95 % CI)^1^ | *p* | HR (95 % CI)^1^ | *p* | HR (95 % CI)^1^ | *p* | HR (95 % CI)^1^ | *p* |
| 5 E% carbohydrates | 0.85 (0.82, 0.89) | <0.001 | 0.86 (0.80, 0.93) | <0.001 | 0.88 (0.79, 0.97) | 0.013 | 0.87 (0.77, 0.98) | 0.026 |
| 5 E% protein | 0.93 (0.89, 0.98) | 0.007 | 0.91 (0.83, 1.00) | 0.044 | 0.91 (0.80, 1.03) | 0.136 | 0.91 (0.79, 1.06) | 0.250 |
| 5 E% MUFA | 0.76 (0.67, 0.87) | <0.001 | 0.73 (0.58, 0.92) | 0.007 | 0.78 (0.57, 1.07) | 0.122 | 0.73 (0.50, 1.07) | 0.111 |
| 5 E% PUFA | 1.00 (0.97, 1.04) | 0.916 | 1.03 (0.97, 1.10) | 0.284 | 0.96 (0.89, 1.05) | 0.388 | 0.98 (0.88, 1.08) | 0.623 |
| 5 E% linoleic acid (C18:2n-6) | 0.90 (0.86, 0.95) | <0.001 | 0.99 (0.90, 1.08) | 0.756 | 0.90 (0.79, 1.03) | 0.117 | 0.89 (0.76, 1.04) | 0.151 |
| 1 E% n-3 FA | 1.09 (1.05, 1.13) | <0.001 | 1.05 (0.98, 1.11) | 0.146 | 1.05 (0.96, 1.14) | 0.259 | 1.07 (0.97, 1.19) | 0.182 |

AMI: Acute myocardial infarction. CVD: cardiovascular disease. IHD: ischaemic heart disease. MUFA: monounsaturated fatty acids, PUFA: polyunsaturated fatty acids ^1^ Cox proportional hazards regression stratified by birth cohort (<1930, 1930-34, 1935-39, 1940-44, 1945-49, 1950-54, and ≥1955) with age as timescale, adjusted for sex, energy intake, body mass index, physical activity, smoking habits, attained education, marital status, self-reported comorbidities (i.e., history of myocardial infarction, stroke, angina or diabetes, treatment for high blood pressure or use of nitro-glycerine), and energy from other types of fat, carbohydrates and protein (depending on type of substitution), and cholesterol (in mg/1000 kcal).

# Supplementary Table 7. Pearson correlation coefficients between cumulative average intakes of individual SFA.

|  | 4:0-10:0 | 12:0 | 14:0 | 16:0 | 18:0 | 12:0-16:0 | 12:0-18:0 |
| --- | --- | --- | --- | --- | --- | --- | --- |
| SFA^1^ |  |  |  |  |  |  |  |
| 4:0-10:0 | 1 |  |  |  |  |  |  |
| 12:0 | 0.73 | 1 |  |  |  |  |  |
| 14:0 | 0.92 | 0.86 | 1 |  |  |  |  |
| 16:0 | 0.79 | 0.73 | 0.85 | 1 |  |  |  |
| 18:0 | 0.66 | 0.65 | 0.69 | 0.93 | 1 |  |  |
| 12-16:0 | 0.85 |  |  |  | 0.86 | 1 |  |
| 12-18:0 | 0.83 |  |  |  |  |  | 1 |

^1^ C4:0-C10:0 = sum of butyric acid, caproic acid, caprylic acid and capric acid; C12:0 = lauric acid; C14:0 = myristic acid; C16:0 = palmitic acid; C18:0 = stearic acid.

# Supplementary Table 8. Association between intake of individual saturated fatty acids and total and cause-specific mortality.

| Quintile of intake | 1 | 2 | 3 | 4 | 5 | *p*_trend_ |
| --- | --- | --- | --- | --- | --- | --- |
| ***C4:0-10:0^1^*** | | | | | | |
| Median intake, E% | 0.7 | 1.0 | 1.3 | 1.6 | 2.1 |  |
| ***Total mortality*** | | | | | | |
| N cases/ person-years | 5796/ 545305.67 | 5771/  538040.00 | 5827/  519510.25 | 5742/  482259.58 | 5419/ 404249.17 |  |
| Sex-adjusted, HR (95% CI)^2^ | 1 (ref.) | 0.95 (0.91, 0.98) | 0.98 (0.94, 1.02) | 1.03 (0.99, 1.06) | 1.07 (1.03, 1.11) | <0.001 |
| Multivariable-adjusted, HR (95% CI)^3^ | 1 | 0.97 (0.93, 1.00) | 1.00 (0.97, 1.04) | 1.05 (1.00, 1.09) | 1.12 (1.06, 1.18) | <0.001 |
| ***CVD mortality*** | | | | | | |
| N deaths/person-years | 1924/ 545305.67 | 1956/ 538040.00 | 1812/ 519510.25 | 1855/ 482259.58 | 1771/ 404249.17 |  |
| Sex-adjusted, HR (95% CI)^2^ | 1 | 0.96 (0.90, 1.02) | 0.90 (0.85, 0.96) | 0.96 (0.90, 1.03) | 0.96 (0.90, 1.02) | 0.258 |
| Multivariable-adjusted, HR (95% CI)^3^ | 1 | 1.00 (0.94, 1.07) | 0.97 (0.90, 1.03) | 1.03 (0.96, 1.11) | 1.11 (1.01, 1.21) | 0.036 |
| ***IHD mortality*** | | | | | | |
| N cases/person-years | 943/ 545305.67 | 982/ 538040.00 | 932/ 519510.25 | 926/ 482259.58 | 948/ 404249.17 |  |
| Sex-adjusted, HR (95% CI)^2^ | 1 | 1.00 (0.91, 1.09) | 0.96 (0.87, 1.05) | 0.98 (0.89, 1.07) | 1.02 (0.93, 1.12) | 0.820 |
| Multivariable-adjusted, HR (95% CI)^3^ | 1 | 1.05 (0.96, 1.15) | 1.04 (0.95, 1.15) | 1.06 (0.95, 1.18) | 1.21 (1.06, 1.38) | 0.011 |
| ***AMI mortality*** | | | | | | |
| N cases/person-years | 666/ 545305.67 | 670/ 538040.00 | 631/ 519510.25 | 649/ 482259.58 | 666/ 404249.17 |  |
| Sex-adjusted, HR (95% CI)^2^ | 1 | 0.97 (0.87, 1.07) | 0.92 (0.82, 1.02) | 0.97 (0.87, 1.08) | 1.02 (0.91, 1.13) | 0.789 |
| Multivariable-adjusted, HR (95% CI)^3^ | 1 | 1.02 (0.91, 1.14) | 1.01 (0.90, 1.13) | 1.07 (0.95, 1.21) | 1.24 (1.06, 1.46) | 0.009 |
| ***C12:0^1^*** | | | | | | |
| Median intake, E% | 0.4 | 0.5 | 0.5 | 0.6 | 0.8 |  |
| ***Total mortality*** | | | | | | |
| N cases/ person-years | 5776/ 578809.42 | 5787/ 543683.50 | 5923/ 508178.50 | 5770/ 468889.08 | 5299/ 389804.17 |  |
| Sex-adjusted, HR (95% CI)^2^ | 1 (ref.) | 0.98 (0.94, 1.01) | 1.04 (1.00, 1.08) | 1.07 (1.03, 1.11) | 1.13 (1.09, 1.18) | <0.001 |
| Multivariable-adjusted, HR (95% CI)^3^ | 1 | 0.99 (0.96, 1.03) | 1.06 (1.02, 1.10) | 1.08 (1.03, 1.13) | 1.17 (1.11, 1.23) | <0.001 |
| ***CVD mortality*** | | | | | | |
| N deaths/person-years | 1931/ 578809.42 | 1854/ 543683.50 | 1951/ 508178.50 | 1850/ 468889.08 | 1732/ 389804.17 |  |
| Sex-adjusted, HR (95% CI)^2^ | 1 | 0.92 (0.87, 0.98) | 1.00 (0.93, 1.06) | 0.98 (0.92, 1.05) | 1.01 (0.94, 1.08) | 0.277 |
| Multivariable-adjusted, HR (95% CI)^3^ | 1 | 0.96 (0.90, 1.02) | 1.05 (0.98, 1.12) | 1.03 (0.96, 1.12) | 1.11 (1.01, 1.22) | 0.008 |
| ***IHD mortality*** | | | | | | |
| N cases/person-years | 1000/ 578809.42 | 909/ 543683.50 | 997/ 508178.50 | 955/ 468889.08 | 870/ 389804.17 |  |
| Sex-adjusted, HR (95% CI)^2^ | 1 | 0.89 (0.82, 0.98) | 1.02 (0.93, 1.11) | 1.03 (0.94, 1.12) | 0.99 (0.91, 1.0) | 0.235 |
| Multivariable-adjusted, HR (95% CI)^3^ | 1 | 0.93 (0.85, 1.02) | 1.07 (0.97, 1.18) | 1.06 (0.96, 1.18) | 1.08 (0.95, 1.22) | 0.067 |
| ***AMI mortality*** | | | | | | |
| N cases/person-years | 705/ 578809.42 | 629/ 543683.50 | 683/ 508178.50 | 665/ 468889.08 | 600/ 389804.17 |  |
| Sex-adjusted, HR (95% CI)^2^ | 1 | 0.88 (0.79, 0.97) | 0.98 (0.88, 1.09) | 1.00 (0.90, 1.12) | 0.96 (0.86, 1.07) | 0.651 |
| Multivariable-adjusted, HR (95% CI)^3^ | 1 | 0.90 (0.81, 1.01) | 1.02 (0.91, 1.15) | 1.03 (0.91, 1.17) | 1.02 (0.87, 1.19) | 0.375 |
| ***C14:0^1^*** | | | | | | |
| Median intake, E% | 1.3 | 1.6 | 1.8 | 2.1 | 2.6 |  |
| ***Total mortality*** | | | | | | |
| N cases/ person-years | 5696/  582164.25 | 5911/  549192.67 | 5995/  515788.25 | 5696/  465845.75 | 5257/  376373.75 |  |
| Sex-adjusted, HR (95% CI)^2^ | 1 (ref.) | 1.00 (0.96, 1.03) | 1.05 (1.01, 1.09) | 1.09 (1.05, 1.13) | 1.18 (1.13, 1.22) | <0.001 |
| Multivariable-adjusted, HR (95% CI)^3^ | 1 | 1.01 (0.97, 1.05) | 1.04 (1.00, 1.08) | 1.10 (1.06, 1.15) | 1.22 (1.15, 1.28) | <0.001 |
| ***CVD mortality*** | | | | | | |
| N deaths/person-years | 1875/ 582164.25 | 1971/ 549192.67 | 1940/ 515788.25 | 1803/ 465845.75 | 1729/ 376373.75 |  |
| Sex-adjusted, HR (95% CI)^2^ | 1 | 0.99 (0.93, 1.06) | 1.00 (0.94, 1.07) | 1.00 (0.93, 1.06) | 1.05 (0.99, 1.12) | 0.147 |
| Multivariable-adjusted, HR (95% CI)^3^ | 1 | 1.02 (0.96, 1.09) | 1.02 (0.95, 1.09) | 1.05 (0.97, 1.13) | 1.17 (1.06, 1.28) | 0.002 |
| ***IHD mortality*** | | | | | | |
| N cases/person-years | 958/ 582164.25 | 969/ 549192.67 | 976/ 515788.25 | 912/ 465845.75 | 916/ 376373.75 |  |
| Sex-adjusted, HR (95% CI)^2^ | 1 | 0.98 (0.89, 1.07) | 1.02 (0.93, 1.11) | 1.01 (0.92, 1.11) | 1.08 (0.99, 1.19) | 0.066 |
| Multivariable-adjusted, HR (95% CI)^3^ | 1 | 1.00 (0.92, 1.10) | 1.03 (0.94, 1.14) | 1.05 (0.95, 1.17) | 1.19 (1.05, 1.35) | 0.009 |
| ***AMI mortality*** | | | | | | |
| N cases/person-years | 676/ 582164.25 | 665/ 549192.67 | 669/ 515788.25 | 624/ 465845.75 | 648/ 376373.75 |  |
| Sex-adjusted, HR (95% CI)^2^ | 1 | 0.95 (0.85, 1.06) | 0.98 (0.88, 1.09) | 0.97 (0.87, 1.09) | 1.07 (0.96, 1.20) | 0.174 |
| Multivariable-adjusted, HR (95% CI)^3^ | 1 | 0.98 (0.88, 1.09) | 1.01 (0.90, 1.13) | 1.02 (0.90, 1.16) | 1.20 (1.03, 1.40) | 0.021 |
| ***C16:0^1^*** | | | | | | |
| Median intake, E% | 5.8 | 6.6 | 7.2 | 7.8 | 8.8 |  |
| ***Total mortality*** | | | | | | |
| N cases/ person-years | 5994/ 574377.08 | 5948/  540035.83 | 5923/  512469.42 | 5566/  470605.83 | 5124/  391876.50 |  |
| Sex-adjusted, HR (95% CI)^2^ | 1 (ref.) | 1.05 (1.01, 1.09) | 1.12 (1.08, 1.16) | 1.19 (1.15, 1.23) | 1.33 (1.28, 1.38) | <0.001 |
| Multivariable-adjusted, HR (95% CI)^3^ | 1 | 1.02 (0.98, 1.07) | 1.08 (1.03, 1.13) | 1.11 (1.05, 1.17) | 1.24 (1.16, 1.34) | <0.001 |
| ***CVD mortality*** | | | | | | |
| N deaths/person-years | 2034/ 574377.08 | 1963/ 540035.83 | 1929/ 512469.42 | 1743/ 470605.83 | 1649/ 391876.50 |  |
| Sex-adjusted, HR (95% CI)^2^ | 1 | 1.02 (0.96, 1.08) | 1.07 (1.01, 1.14) | 1.09 (1.02, 1.16) | 1.20 (1.12, 1.28) | <0.001 |
| Multivariable-adjusted, HR (95% CI)^3^ | 1 | 1.02 (0.95, 1.10) | 1.07 (0.99, 1.17) | 1.07 (0.97, 1.18) | 1.24 (1.09, 1.40) | 0.002 |
| ***IHD mortality*** | | | | | | |
| N cases/person-years | 1022/ 574377.08 | 968/ 540035.83 | 988/ 512469.42 | 893/ 470605.83 | 860/ 391876.50 |  |
| Sex-adjusted, HR (95% CI)^2^ | 1 | 1.01 (0.93, 1.10) | 1.11 (1.02, 1.21) | 1.13 (1.03, 1.23) | 1.23 (1.12, 1.35) | <0.001 |
| Multivariable-adjusted, HR (95% CI)^3^ | 1 | 1.04 (0.94, 1.15) | 1.15 (1.02, 1.29) | 1.15 (1.01, 1.32) | 1.33 (1.12, 1.59) | 0.001 |
| ***AMI mortality*** | | | | | | |
| N cases/person-years | 721/ 574377.08 | 664/ 540035.83 | 674/ 512469.42 | 629/ 470605.83 | 594/ 391876.50 |  |
| Sex-adjusted, HR (95% CI)^2^ | 1 | 0.98 (0.88, 1.09) | 1.07 (0.96, 1.19) | 1.11 (1.01, 1.24) | 1.19 (1.06, 1.32) | <0.001 |
| Multivariable-adjusted, HR (95% CI)^3^ | 1 | 1.00 (0.89, 1.13) | 1.09 (0.95, 1.25) | 1.12 (0.96, 1.32) | 1.26 (1.02, 1.56) | 0.021 |
| ***C18:0^1^*** | | | | | | |
| Median intake, E% | 2.9 | 3.3 | 3.6 | 3.9 | 4.3 |  |
| ***Total mortality*** | | | | | | |
| N cases/ person-years | 5968/  569980.17 | 5931/  527826.42 | 5846/  501392.92 | 5533/  475555.08 | 5277/  414610.08 |  |
| Sex-adjusted, HR (95% CI)^2^ | 1 (ref.) | 1.04 (1.01, 1.08) | 1.08 (1.04, 1.12) | 1.11 (1.07, 1.15) | 1.33 (1.28, 1.38) | <0.001 |
| Multivariable-adjusted, HR (95% CI)^3^ | 1 | 1.03 (0.98, 1.08) | 1.05 (0.99, 1.10) | 1.04 (0.97, 1.11) | 1.18 (1.08, 1.29) | <0.001 |
| ***CVD mortality*** | | | | | | |
| N deaths/person-years | 2012/ 569980.17 | 1985/ 527826.42 | 1855/ 501392.92 | 1784/ 475555.08 | 1682/ 414610.08 |  |
| Sex-adjusted, HR (95% CI)^2^ | 1 | 1.02 (0.96, 1.09) | 1.00 (0.94, 1.07) | 1.04 (0.97, 1.11) | 1.20 (1.13, 1.28) | <0.001 |
| Multivariable-adjusted, HR (95% CI)^3^ | 1 | 1.03 (0.96, 1.12) | 1.00 (0.91, 1.10) | 1.03 (0.92, 1.15) | 1.16 (1.00, 1.34) | 0.104 |
| ***IHD mortality*** | | | | | | |
| N cases/person-years | 1009/ 569980.17 | 975/ 527826.42 | 952/ 501392.92 | 932/ 475555.08 | 863/ 414610.08 |  |
| Sex-adjusted, HR (95% CI)^2^ | 1 | 1.01 (0.92, 1.10) | 1.03 (0.94, 1.12) | 1.08 (0.98, 1.18) | 1.19 (1.08, 1.30) | <0.001 |
| Multivariable-adjusted, HR (95% CI)^3^ | 1 | 1.02 (0.91, 1.13) | 1.02 (0.90, 1.17) | 1.07 (0.91, 1.25) | 1.13 (0.91, 1.39) | 0.253 |
| ***AMI mortality*** | | | | | | |
| N cases/person-years | 692/ 569980.17 | 686/ 527826.42 | 663/ 501392.92 | 651/ 475555.08 | 590/ 414610.08 |  |
| Sex-adjusted, HR (95% CI)^2^ | 1 | 1.03 (0.93, 1.14) | 1.04 (0.94, 1.16) | 1.09 (0.98, 1.21) | 1.17 (1.05, 1.30) | 0.003 |
| Multivariable-adjusted, HR (95% CI)^3^ | 1 | 1.05 (0.92, 1.20) | 1.06 (0.90, 1.24) | 1.11 (0.92, 1.34) | 1.15 (0.90, 1.48) | 0.258 |

AMI: Acute myocardial infarction. CVD: cardiovascular disease. IHD: ischaemic heart disease. ^1^ C4:0-C10:0 = sum of butyric acid, caproic acid, caprylic acid and capric acid; C12:0 = lauric acid; C14:0 = myristic acid; C16:0 = palmitic acid; C18:0 = stearic acid. E% = percentage of energy. IQR = inter-quartile range.
^2^ Cox proportional hazards regression models were stratified by birth cohort (<1930, 1930-34, 1935-39, 1940-44, 1945-49, 1950-54, and ≥1955) with age as timescale.
^3^ Further adjusted energy intake, body mass index, physical activity, smoking habits, attained education, marital status, self-reported comorbidities (i.e., history of myocardial infarction, stroke, angina or diabetes, treatment for high blood pressure or use of nitro-glycerine), and energy from other types of fat and protein (in E%), and cholesterol (in mg/1000 kcal); the HRs in this model can be interpreted as the associations with intakes of individual SFA at the expense of carbohydrates.

# Supplementary Table 9. Hazard ratios (95 % CI) for total, CVD, IHD and AMI mortality associated with isocaloric replacement of energy from C12:0-C18:0 with other macronutrients.

| Substitution | Total mortality | | CVD mortality | | IHD mortality | | MI mortality | |
| --- | --- | --- | --- | --- | --- | --- | --- | --- |
|  | HR (95 % CI)^1^ | *p* | HR (95 % CI)^1^ | *p* | HR (95 % CI)^1^ | *p* | HR (95 % CI)^1^ | *p* |
| 5 E% carbohydrates | 0.71 (0.62, 0.82) | <0.001 | 0.84 (0.66, 1.08) | 0.176 | 0.82 (0.58, 1.16) | 0.262 | 0.94 (0.62, 1.42) | 0.776 |
| 5 E% protein | 0.76 (0.66, 0.89) | 0.001 | 0.88 (0.67, 1.14) | 0.337 | 0.84 (0.58, 1.21) | 0.357 | 0.99 (0.64, 1.53) | 0.952 |
| 5 E% MUFA | 0.56 (0.43, 0.73) | <0.001 | 0.71 (0.45, 1.12) | 0.141 | 0.69 (0.36, 1.30) | 0.250 | 0.83 (0.39, 1.79) | 0.640 |
| 5 E% PUFA | 0.84 (0.74, 0.96) | 0.009 | 0.99 (0.79, 1.25) | 0.953 | 0.91 (0.66, 1.24) | 0.543 | 1.04 (0.71, 1.53) | 0.820 |
| 5 E% linoleic acid (C18:2n-6) | 0.84 (0.74, 0.96) | 0.011 | 1.00 (0.79, 1.25) | 0.973 | 0.91 (0.66, 1.25) | 0.564 | 1.05 (0.72, 1.54) | 0.789 |
| 1 E% n-3 FA | 1.05 (1.00, 1.11) | 0.066 | 1.04 (0.94, 1.14) | 0.457 | 1.04 (0.91, 1.18) | 0.550 | 1.12 (0.96, 1.31) | 0.157 |

AMI: Acute myocardial infarction. CVD: cardiovascular disease. IHD: ischaemic heart disease. MUFA: monounsaturated fatty acids, PUFA: polyunsaturated fatty acids.
^1^ Cox proportional hazards regression stratified by birth cohort (<1930, 1930-34, 1935-39, 1940-44, 1945-49, 1950-54, and ≥1955) with age as timescale, adjusted for other individual saturated fatty acids and the same covariates as in Supplementary Table 6.

# Supplementary Table 10. Hazard ratios (95 % confidence intervals) for the highest quintile (Q5) of cumulative average intake of saturated fatty acids (SFA), and per 5 E% from SFA, excluding the first 2 years of follow-up.

|  | Q5 vs. Q1 | Per 5 E% |
| --- | --- | --- |
|  | HR (95% CI)^1^ | HR (95% CI) |
| Total mortality (28,320 deaths) | 1.26 (1.18, 1.34) | 1.18 (1.13, 1.23), *p* <0.001 |
| CVD mortality (9233 deaths) | 1.25 (1.12, 1.39) | 1.16 (1.07, 1.25), *p* <0.001 |
| IHD mortality (4676 deaths) | 1.32 (1.04, 1.33) | 1.15 (1.03, 1.27), *p* = 0.010 |
| AMI mortality (3235 deaths) | 1.35 (1.12, 1.62) | 1.17 (1.03, 1.32), *p* = 0.015 |

AMI: Acute myocardial infarction. CVD: cardiovascular disease. IHD: ischaemic heart disease.
^1^ Cox proportional hazards regression stratified by birth cohort (<1930, 1930-34, 1935-39, 1940-44, 1945-49, 1950-54, and ≥1955) with age as timescale, adjusted for energy intake, body mass index, physical activity, smoking habits, attained education, marital status, self-reported comorbidities (i.e., history of myocardial infarction, stroke, angina or diabetes, treatment for high blood pressure or use of nitro-glycerine), and energy from other types of fat and protein (in E%) and cholesterol (in mg/1000 kcal); the HRs in this model can be interpreted as the associations with intakes of SFA at the expense of carbohydrates.

# Supplementary Table 11. Hazard ratios (95 % confidence intervals) for the highest quintile (Q5) of cumulative average intake of saturated fatty acids (SFA), and per 5 E% from SFA during the first and second half of the follow-up period (22.5 years).

|  | 1^st^ half | | 2^nd^ half | |
| --- | --- | --- | --- | --- |
|  | Q5 vs. Q1 | Per 5 E% | Q5 vs. Q1 | Per 5 E% |
|  | HR (95% CI)^1^ | | HR (95% CI)^1^ | |
| Total mortality | 1.28 (1.14, 1.43) | 1.18 (1.10, 1.27)  *p* <0.001 | 1.23 (1.14, 1.33) | 1.17 (1.11, 1.23)  *p* <0.001 |
| CVD mortality | 1.34 (1.11, 1.62) | 1.22 (1.07, 1.38)  *p* = 0.002 | 1.18 (1.03, 1.35) | 1.11 (1.02, 1.22)  *p* = 0.022 |
| IHD mortality | 1.38 (1.09, 1.75) | 1.22 (1.04, 1.43)  *p* = 0.013 | 1.25 (1.02, 1.53) | 1.07 (0.93, 1.23)  *p* = 0.357 |
| AMI mortality | 1.25 (0.95, 1.64) | 1.17 (0.97, 1.40)  *p* = 0.101 | 1.38 (1.08, 1.77) | 1.13 (0.95, 1.34)  *p* = 0.162 |

AMI: Acute myocardial infarction. CVD: cardiovascular disease. IHD: ischaemic heart disease.
^1^ Cox proportional hazards regression stratified by birth cohort (<1930, 1930-34, 1935-39, 1940-44, 1945-49, 1950-54, and ≥1955) with age as timescale, adjusted for energy intake, body mass index, physical activity, smoking habits, attained education, marital status, self-reported comorbidities (i.e., history of myocardial infarction, stroke, angina or diabetes, treatment for high blood pressure or use of nitro-glycerine), and energy from other types of fat and protein (in E%) and cholesterol (in mg/1000 kcal); the HRs in this model can be interpreted as the associations with intakes of SFA at the expense of carbohydrates.

# Supplementary Table 12. Hazard ratios (95 % confidence intervals) for the highest quintile (Q5) of cumulative average intake of saturated fatty acids (SFA), and per 5 E% from SFA excluding individuals reporting to follow a special diet (n = 1900) during the screenings.

|  | Q5 vs. Q1 | Per 5 E% |
| --- | --- | --- |
|  | HR (95% CI)^1^ | HR (95% CI)^1^ |
| Total mortality | 1.27 (1.19, 1.35) | 1.18 (1.13, 1.24), *p* <0.001 |
| CVD mortality | 1.27 (1.13, 1.42) | 1.17 (1.08, 1.26), *p* <0.001 |
| IHD mortality | 1.35 (1.15, 1.58) | 1.16 (1.04, 1.29), *p* = 0.010 |
| AMI mortality | 1.38 (1.14, 1.66) | 1.17 (1.02, 1.33), *p* = 0.021 |

AMI: Acute myocardial infarction. CVD: cardiovascular disease. IHD: ischaemic heart disease.
^1^ Cox proportional hazards regression stratified by birth cohort (<1930, 1930-34, 1935-39, 1940-44, 1945-49, 1950-54, and ≥1955) with age as timescale, adjusted for energy intake, body mass index, physical activity, smoking habits, attained education, marital status, self-reported comorbidities (i.e., history of myocardial infarction, stroke, angina or diabetes, treatment for high blood pressure or use of nitro-glycerine), and energy from other types of fat and protein (in E%) and cholesterol (in mg/1000 kcal); the HRs in this model can be interpreted as the associations with intakes of SFA at the expense of carbohydrates.

# Supplementary Table 13. Hazard ratios (95 % confidence intervals) for the highest quintile (Q5) of cumulative average intake of saturated fatty acids (SFA), and per 5 E% from SFA excluding individuals with reported presence of comorbidities (previous myocardial infarction or stroke, history of angina or diabetes, treatment for high blood pressure or use of nitro-glycerine).

|  | Q5 vs. Q1 | Per 5 E% |
| --- | --- | --- |
|  | HR (95% CI) | HR (95% CI) |
| Total mortality (28,609 deaths) | 1.26 (1.18, 1.34) | 1.18 (1.13, 1.23), *p* <0.001 |
| CVD mortality (9344 deaths) | 1.25 (1.12, 1.40) | 1.17 (1.08, 1.26), *p* <0.001 |
| IHD mortality (4745 deaths) | 1.32 (1.14, 1.54) | 1.16 (1.04, 1.28), *p* = 0.006 |
| MI mortality (3286 deaths) | 1.32 (1.10, 1.59) | 1.16 (1.03, 1.32), *p* = 0.018 |

AMI: Acute myocardial infarction. CVD: cardiovascular disease. IHD: ischaemic heart disease.
^1^ Cox proportional hazards regression stratified by birth cohort (<1930, 1930-34, 1935-39, 1940-44, 1945-49, 1950-54, and ≥1955) with age as timescale, adjusted for energy intake, body mass index, physical activity, smoking habits, attained education, marital status, self-reported comorbidities (i.e., history of myocardial infarction, stroke, angina or diabetes, treatment for high blood pressure or use of nitro-glycerine), and energy from other types of fat and protein (in E%) and cholesterol (in mg/1000 kcal); the HRs in this model can be interpreted as the associations with intakes of SFA at the expense of carbohydrates.

# Supplementary Table 14. Hazard ratios (95 % confidence intervals) for the highest quintile (Q5) of cumulative average intake of saturated fatty acids (SFA), and per 5 E% from SFA when not adjusting for education (n = 79,681).

|  | Q5 vs. Q1 | Per 5 E% |
| --- | --- | --- |
|  | HR (95% CI)^1^ | HR (95% CI)^1^ |
| Total mortality (28,734 deaths) | 1.27 (1.19, 1.35) | 1.18 (1.13, 1.23), *p* <0.001 |
| CVD mortality (9357 deaths) | 1.27 (1.14, 1.41) | 1.16 (1.08, 1.25), *p* <0.001 |
| IHD mortality (4753 deaths) | 1.33 (1.14, 1.55) | 1.14 (1.03, 1.27), *p* = 0.012 |
| AMI mortality (3298 deaths) | 1.33 (1.11, 1.59) | 1.15 (1.01, 1.30), *p* = 0.030 |

AMI: Acute myocardial infarction. CVD: cardiovascular disease. IHD: ischaemic heart disease.
^1^ Cox proportional hazards regression stratified by birth cohort (<1930, 1930-34, 1935-39, 1940-44, 1945-49, 1950-54, and ≥1955) with age as timescale, adjusted for energy intake, body mass index, physical activity, smoking habits, attained education, marital status, self-reported comorbidities (i.e., history of myocardial infarction, stroke, angina or diabetes, treatment for high blood pressure or use of nitro-glycerine), and energy from other types of fat and protein (in E%) and cholesterol (in mg/1000 kcal); the HRs in this model can be interpreted as the associations with intakes of SFA at the expense of carbohydrates.

# Supplementary Table 15. Hazard ratios (95 % confidence intervals) for the highest quintile (Q5) of cumulative average intake of saturated fatty acids (SFA), and per 5 E% from SFA when further adjusting for potassium and vitamin C intake (n = 73,045).

|  | Q5 vs. Q1 | Per 5 E% |
| --- | --- | --- |
|  | HR (95% CI)^1^ | HR (95% CI)^1^ |
| Total mortality (26,352 deaths) | 1.21 (1.13, 1.29) | 1.15 (1.10, 1.21), *p* <0.001 |
| CVD mortality (8555 deaths) | 1.17 (1.04, 1.31) | 1.11 (1.02, 1.20), *p* = 0.01 |
| IHD mortality (4339 deaths) | 1.27 (1.08, 1.50) | 1.12 (1.00, 1.25), *p* = 0.05 |
| AMI mortality (3002 deaths) | 1.26 (1.03, 1.52) | 1.11 (0.97, 1.27), *p* = 0.12 |

AMI: Acute myocardial infarction. CVD: cardiovascular disease. IHD: ischaemic heart disease.
^1^ Cox proportional hazards regression stratified by birth cohort (<1930, 1930-34, 1935-39, 1940-44, 1945-49, 1950-54, and ≥1955) with age as timescale, adjusted for energy intake, body mass index, physical activity, smoking habits, attained education, marital status, self-reported comorbidities (i.e., history of myocardial infarction, stroke, angina or diabetes, treatment for high blood pressure or use of nitro-glycerine), and energy from other types of fat and protein (in E%), cholesterol (in mg/1000 kcal), vitamin C (mg/1000 kcal) and potassium (mg/1000 kcal); the HRs in this model can be interpreted as the associations with intakes of SFA at the expense of carbohydrates.

# Supplementary Table 16. Hazard ratios (95 % confidence intervals) for the highest quintile (Q5) of cumulative average intake of saturated fatty acids (SFA), and per 5 E% from SFA using only baseline nutrient data as exposure.

|  | Q5 vs. Q1 | 5 E% |
| --- | --- | --- |
|  | HR (95% CI) | HR (95% CI) |
| Total mortality | 1.13 (1.07, 1.20) | 1.11 (1.07, 1.15), *p* <0.001 |
| CVD mortality | 1.08 (0.97, 1.19) | 1.09 (1.03, 1.16), *p* = 0.004 |
| IHD mortality | 1.08 (0.93, 1.24) | 1.07 (0.99, 1.17), *p* = 0.103 |
| AMI mortality | 1.11 (0.93, 1.32) | 1.08 (0.97, 1.19), *p* = 0.156 |

AMI: Acute myocardial infarction. CVD: cardiovascular disease. IHD: ischaemic heart disease.
^1^ Cox proportional hazards regression stratified by birth cohort (<1930, 1930-34, 1935-39, 1940-44, 1945-49, 1950-54, and ≥1955) with age as timescale, adjusted for energy intake, body mass index, physical activity, smoking habits, attained education, marital status, self-reported comorbidities (i.e., history of myocardial infarction, stroke, angina or diabetes, treatment for high blood pressure or use of nitro-glycerine), and energy from other types of fat and protein (in E%) and cholesterol (in mg/1000 kcal); the HRs in this model can be interpreted as the associations with intakes of SFA at the expense of carbohydrates.

**S table 15.** Hazard ratios (95 % confidence intervals) per 1 E% change in saturated fatty acid intake between screenings (n = 42,507).

|  | >1 E% increase vs. no change | Per 1 E% |
| --- | --- | --- |
| Total mortality | 1.06 (1.02, 1.11) | 0.98 (0.94, 1.03), *p* = 0.47 |
| CVD mortality | 1.04 (0.96, 1.12) | 0.97 (0.90, 1.04), *p* = 0.42 |
| IHD mortality | 1.02 (0.91, 1.14) | 0.98 (0.89, 1.08), *p* = 0.71 |
| AMI mortality | 1.01 (0.89, 1.16) | 0.98 (0.87, 1.11), *p* = 0.80 |
| Med oppfølging t.o.m. 31.12.1988 |  |  |
| Total mortality (746 deaths) | 1.27 (1.02, 1.56) | 1.00 (0.96, 1.04), *p* = 0.98 |
| CVD mortality (311 deaths) | 1.42 (1.02, 1.98) | 0.98 (0.92, 1.04), *p* = 0.48 |
| IHD mortality (216 deaths) | 1.22 (0.82, 1.80) | 1.00 (0.92, 1.07), *p* = 0.93 |
| AMI mortality (181 deaths) | 1.30 (0.84, 2.01) | 0.98 (0.90, 1.06), *p* = 0.66 |

AMI: Acute myocardial infarction. CVD: cardiovascular disease. IHD: ischaemic heart disease.

Cox proportional hazards regression stratified by birth cohort (<1930, 1930-34, 1935-39, 1940-44, 1945-49, 1950-54, and ≥1955) with age as timescale, adjusted for changes in energy intake, body mass index, physical activity, smoking habits, attained education, marital status, self-reported comorbidities (i.e., history of myocardial infarction, stroke, angina or diabetes, treatment for high blood pressure or use of nitro-glycerine), and changes in energy from other types of fat and protein (in E%) and cholesterol (in mg/1000 kcal); the HRs in this model can be interpreted as the associations with a change in intake of SFA at the expense of carbohydrates.
